# Latitudinal Environmental Niches and Riverine Barriers Shaped the Phylogeography of the Central Chilean Endemic *Dioscorea humilis* (Dioscoreaceae)

**DOI:** 10.1371/journal.pone.0110029

**Published:** 2014-10-08

**Authors:** Juan Viruel, Pilar Catalán, José Gabriel Segarra-Moragues

**Affiliations:** 1 Departamento de Agricultura y Economía Agraria, Escuela Politécnica Superior de Huesca, Universidad de Zaragoza, Huesca, Spain; 2 Department of Botany, Institute of Biology, Tomsk State University, Tomsk, Russia; 3 Departamento de Ecología, Centro de Investigaciones sobre Desertificación (CIDE), Consejo Superior de Investigaciones Científicas (CSIC), Moncada, Valencia, Spain; National Cheng-Kung University, Taiwan

## Abstract

The effects of Pleistocene glaciations and geographical barriers on the phylogeographic patterns of lowland plant species in Mediterranean-climate areas of Central Chile are poorly understood. We used *Dioscorea humilis* (Dioscoreaceae), a dioecious geophyte extending 530 km from the Valparaíso to the Bío-Bío Regions, as a case study to disentangle the spatio-temporal evolution of populations in conjunction with latitudinal environmental changes since the Last Inter-Glacial (LIG) to the present. We used nuclear microsatellite loci, chloroplast (cpDNA) sequences and environmental niche modelling (ENM) to construct current and past scenarios from bioclimatic and geographical variables and to infer the evolutionary history of the taxa. We found strong genetic differentiation at nuclear microsatellite loci between the two subspecies of *D. humilis*, probably predating the LIG. Bayesian analyses of population structure revealed strong genetic differentiation of the widespread *D. humilis* subsp. *humilis* into northern and southern population groups, separated by the Maipo river. ENM revealed that the ecological niche differentiation of both groups have been maintained up to present times although their respective geographical distributions apparently fluctuated in concert with the climatic oscillations of the Last Glacial Maximum (LGM) and the Holocene. Genetic data revealed signatures of eastern and western postglacial expansion of the northern populations from the central Chilean depression, whereas the southern ones experienced a rapid southward expansion after the LGM. This study describes the complex evolutionary histories of lowland Mediterranean Chilean plants mediated by the summed effects of spatial isolation caused by riverine geographical barriers and the climatic changes of the Quaternary.

## Introduction

Historical, geographical and climatic events have a strong influence on the genetic diversity of species [1]. In South America, most of the biogeographical studies of plants have focused on the effects of Pleistocene glaciations and postglacial climatic fluctuations on Andean species, having identified several lowland refugia [2]–[4]. However, the phylogeography of lowland species inhabiting ice-free areas during glaciations remains scarcely documented. Population genetic diversity and structure of lowland taxa are not expected to have been severely impacted by the direct effect of glaciations because of the absence of ice sheets in the central Chilean depression [5]–[6] and the North-to-South arrangement of the Andes, which allowed latitudinal migration [7]. Additionally, the central Chilean depression and its surrounding coastal areas provided the most suitable and stable environments for the establishment of plant and animal populations during Quaternary glaciations [2], [4], . Unlike the high Andean regions, the areas currently occupied by lowland species likely allowed *in situ* survival during glaciations; however, global temperature cooling during the glaciations could have also contributed to narrowing their geographical ranges to warmer areas.

During the Last Glacial Maximum (LGM, 25000–15000 years ago), ice sheets extended from 56°S to 35°S along the Andes [5]–[6]. These extensive glaciations affected the central Chilean valleys of Maipo and Aconcagua [10]. Although Quaternary glaciers reached down to 1200–2800 m.a.s.l. [10]–[11], their occurrence was coupled with a decrease in temperature and an increase in the precipitation rates at lower altitudes [12]–[13].

In addition to the West-to-East barriers imposed by the Coastal Cordillera and the Andean mountains, it has been proposed that large rivers (e.g. Aconcagua, Maipo) that completely cross Chile may contribute to within-species differentiation [14]. Water volume carried by those rivers fluctuated concomitantly with Pleistocene glaciations, increasing considerably due to ice-melting from the Andes. Accordingly, their potential barrier effect to species migration was stronger during the glacial periods than during the interglacials [15]. The genetic structure of central Chilean lowland species during these glaciations may have been affected by an East-to-West contraction of their distribution ranges towards the central Chilean depression and by their dispersal ability to bypass the transversal river barriers during latitudinal migration.

**Figure 1:**
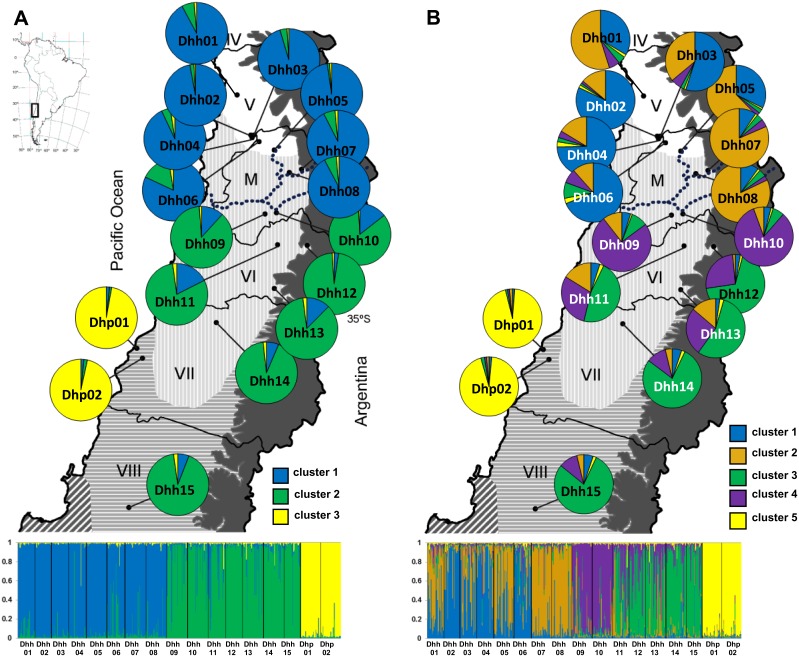
Geographical distribution of sampled populations of *Dioscorea humilis* in Chile (Table 1) and Bayesian analyses of the genetic structure of 15 populations of *D. humilis* subsp. *humilis* and two populations of *D. humilis* subsp. *polyanthes* based on nuclear microsatellite data. The mean proportion of membership of each predefined population to each of the A, three (*K* = 3), and B, five (*K* = 5), most likely inferred genetic clusters is shown. The dotted line indicates the location of the Maipo river. Chilean administrative regions: IV, Coquimbo, V, Valparaíso, M, Metropolitana, VI, Libertador General Bernardo O’Higgins, VII, Maule, and VIII, Bío-Bío. Geographical ranges of five climatic zones in central Chile from Castillo *et al*., [20] are superimposed on the maps. From North to South: semiarid Mediterranean (white), sub-humid Mediterranean (vertical shading), humid Mediterranean (horizontal shading), hyper humid Mediterranean (diagonal shading) and eastern Andean Continental (solid grey). Map contour constructed from spatial data retrieved from http://www.diva-gis.org/gdata.

The *Epipetrum* group of *Dioscorea* is a small evolutionary lineage of the Dioscoreaceae including two species, *D. humilis* Colla and *D. biloba* (Phil.) Caddick & Wilkin, with two subspecies in each [16] that probably originated in the late Miocene (Viruel *et al*., unpublished data). The diversification of this small group followed the retreat of the marine transgressions of the middle Miocene (15-11 Ma) which covered central Chile, providing new lands available for plant colonization from the late Miocene onwards [17]. *Dioscorea humilis* is a dioecious, diploid (2*n* = 14), dwarf geophyte with a widespread distribution spanning five central Chilean regions (530 km), from its northernmost limit in Valparaíso to its southernmost limit in Bío-Bío [16], [18] (Fig. 1, Table 1). Its current distribution range is included within the Mediterranean-type bioclimatic region of Chile [19], which is bounded northwards by the Atacama Desert and southwards by temperate forests [8]. This North-to-South range covers three different climatic environments (Fig. 1): semi-arid, sub-humid and humid Mediterranean climates [20]. *Dioscorea humilis* occurs in the lowland depression between the coastal mountain range and the Andes. It includes two subspecies, the widespread *D. humilis* subsp. *humilis* and the narrow parapatric Maule coastal endemic *D. humilis* subsp. *polyanthes* (F. Phil.) Viruel, Segarra-Moragues & Villar [16] (Fig. 1).

**Table 1:** Population data and genetic diversity indices of 17 Chilean populations of *Dioscorea humilis* for eight nuclear microsatellite loci and overall estimates for taxonomic and geographical population groups.

Code	Locality	Latitude	Longitude	Altitude (m)	*N* 1	A^1^	*H*_O_ 1	*H*_E_ 1	*F*_IS_ 1
***Dioscorea humilis*** ** subsp. ** ***Humilis*** **(Dhh01 to Dhh15)**					488	3.969	0.468	0.425	−0.103***
**Northern ** ***D. humilis*** ** subsp.** ***humilis*** ** (Dhh01 to Dhh08)**					257	3.800	0.466	0.410	−0.136***
**Dhh01**	Valparaíso:Catapilco, rincónde La Mestiza	32° 32′ 33.4″ S	71° 17′ 37.7″ W	90	30	4.125	0.471	0.405	−0.165**
**Dhh02**	Metropolitana:Granizo, ParqueNacional de LaCampana, sectorGranizo	32° 58′ 54.9″ S	71° 07′ 58.2″ W	450	28	4.000	0.424	0.386	−0.100**
**Dhh03**	Metropolitana:Granizo, ParqueNacional de LaCampana, La Troyabridge.	32° 59′ 05.3″ S	71° 08′ 17.8″ W	420	30	4.125	0.425	0.371	−0.148**
**Dhh04**	Metropolitana: ParqueNacional de LaCampana, sectorCajón grande	33° 00′ 06.1″ S	71° 07′ 53.5″ W	340	30	3.500	0.350	0.391	+0.107*
**Dhh05**	Metropolitana:Carretera a Til Til,Entrance to Parcelaciónde El tranque.	33° 08′ 40.7″ S	70° 52′ 34.8″ W	530	36	3.875	0.438	0.392	+0.036**
**Dhh06**	Metropolitana: Roadfrom Til Til toLimache, detour toLa Vega through elCamarico.	33° 03′ 28.3″ S	71° 02′ 57.2″ W	700	31	3.875	0.510	0.458	+0.019**
**Dhh07**	Metropolitana: Santiagode Chile, CerroManquehue, creekstarting from Agua delPalo.	33° 21′ 50.3″ S	70° 34′ 56.9″ W	820	36	3.625	0.523	0.423	−0.241**
**Dhh08**	Metropolitana: Santiago de Chile, Cerro de Renca.	33° 23′ 43.1″ S	70° 42′ 38.9″ W	540	36	4.500	0.560	0.453	−0.135**
**Southern ** ***D. humilis*** ** subsp.** ***humilis*** ** (Dhh09 to Dhh15)**					231	4.163	0.472	0.442	−0.068***
**Dhh09**	Metropolitana: Maipo,Cerro Cantillana, side offorest track from Ragueto Pabellón.	33° 51′ 10.8″ S	70° 58′ 44.5″ W	630	36	4.625	0.460	0.467	+0.108**
**Dhh10**	Metropolitana : Maipo,Cerros de Aculeo.	33° 50′ 04.4″ S	70° 51′ 14.0″ W	380	36	4.714	0.549	0.521	−0.044^ns^
**Dhh11**	ÓHiggins: Road fromRancagua to Doñihue.	34° 11′ 28.2″ S	70° 50′ 39.4″ W	430	30	5.000	0.432	0.459	+0.078**
**Dhh12**	ÓHiggins: Road fromCoya to Pangal.	34° 12′ 03.2″ S	70° 30′ 58.4″ W	850	29	3.250	0.453	0.357	−0.177**
**Dhh13**	ÓHiggins: Road from SanFernando to Tinguiririca,next to La Rufina foresttrack.	34° 40′ 27.0″ S	70° 52′ 56.4″ W	550	36	4.625	0.485	0.444	−0.073**
**Dhh14**	Maule: Road fromCuricó to Sagrada Familia.	35° 03′ 03.0″ S	70° 31′ 03.2″ W	170	36	4.875	0.447	0.413	+0.046^ns^
**Dhh15**	Bio-Bio: BetweenYumbel and Monteáguila	37° 05′ 00.4″ S	72° 28′ 46.1″ W	110	28	3.875	0.556	0.502	−0.064*
***Dioscorea humilis*** ** subsp.** ***polyanthes*** ** (Dhp01-Dhp02)**					**70**	4.131	0.574	0.480	−0.195***
**Dhp01**	Maule: Constitución,just after the river Maulebridge to Putú.	35° 20′ 05.6″ S	72° 23′ 17.2″ W	20	35	4.250	0.598	0.489	−0.222**
**Dhp02**	Maule: de Constitucióna San Javier	35° 26′ 24.1″ S	72° 20′ 00.2″ W	330	35	4.500	0.550	0.475	−0.162^ns^

1 *N*, sample size; *A*, mean number of alleles per locus; *H*_O_, *H*_E_, observed and expected heterozygosity, respectively; *F*_IS_, inbreeding coefficient. Significance: *, *P*<0.05, **, *P*<0.01, ***, *P*<0.001, ns, not significant.

*Dioscorea humilis* has a sprawling habit with shoots creeping among rock crevices. Flowers are tiny and inconspicuous; those of males are produced in pauciflorate racemes, and those of females are generally solitary. The pollination mechanisms are unknown, but flower morphology suggests the implication of a small-sized insect. The wingless seeds are produced in capsules which are sustained by spirally curled peduncles that attach capsules close to the ground or inside rock crevices, suggesting extremely short-distance seed dispersal [16].

We used nuclear microsatellite markers and cpDNA sequences to document the current patterns of population genetic diversity and structure in *D. humilis*. Additionally, Environmental Niche Modelling (ENM) was estimated on the current range extension of the infraspecific genetic groups and projected to two past scenarios, the Last Glacial Maximum (LGM) and Last Inter-Glacial (LIG). Phylogeographical patterns obtained from molecular markers, together with the estimated past variation in range extension, were investigated to elucidate the effect of Pleistocene glaciations and geological and hydrological barriers in the evolutionary history of lowland central Chilean species with limited dispersal abilities like *D. humilis*.

## Materials and Methods

### Ethic Statement

Necessary permits for fieldwork and sampling were obtained from the Corporación Nacional Forestal (CONAF-Chile).

### Plant Sampling, DNA Extraction and Microsatellite Amplification

Fresh leaves from a total 558 individuals from 17 populations of *D. humilis* were collected throughout its entire distribution range. Fifteen populations (Dhh01-Dhh15) corresponded to *D. humilis* subsp. *humilis* and two populations (Dhp01-Dhp02) to *D. humilis* subsp. *polyanthes* (Table 1, Fig. 1). Eight populations of *D. humilis* subsp. *humilis* (Dhh01-Dhh08) were located North of the Maipo river basin, growing in semi-arid Mediterranean-type climate areas, whereas the other seven populations (Dhh09-Dhh15) were located South of it (Table 1, Fig. 1). Five of these (Dhh09-Dhh14) were growing in sub-humid Mediterranean-type climate areas, and the southernmost population (Dhh15), together with two populations of *D. humilis* subsp. *polyanthes* (Dhp01-Dhp02), were growing in humid Mediterranean-type climate areas [20]. DNA extraction followed the procedure described in [21]. Individuals were genotyped for eight unlinked microsatellite following [22].

### Plastid DNA Amplification and Sequencing

Two plastid regions, *trn*T-L and *trn*L-F [23] were amplified and sequenced in up to six individuals per population following [21]. Sequences were deposited in Genbank under the accession numbers KF357945-KF357955. A combined matrix of individual sequences of both plastid regions totalling 54 sequences was used in subsequent analyses.

### Microsatellite Analysis

Allele frequencies and genetic diversity indices were calculated in all populations using GENETIX 4.05 [24]. Deviations from Hardy-Weinberg equilibrium were tested in all populations using GENEPOP v. 4.0 [25]. Different taxonomic and geographical population groups were compared to reveal differences in average values of allelic richness (A*), observed heterozygosity (*H*_O_), genetic diversity within populations (*H*_S_), inbreeding coefficient (*F*_IS_) and population differentiation (*F*_ST_) using FSTAT v. 2.9.3.2 [26], and differences were tested for significance with 10,000-permutation tests. Population pairwise differentiation (*F*_ST_) was calculated with ARLEQUIN 3.11 [27] and tested for significance using 1000 replicates. ARLEQUIN was also used to generate a matrix of pairwise linearized *F*_ST_ values (i.e. *F*_ST_/(1−*F*_ST_); [28]), which was correlated to a log-transformed matrix of geographical distances between populations to test for Isolation By Distance (IBD) through Mantel tests. Significance of correlation was tested with 1000 permutations with NTSYSpc 2.11 [29].

Pairwise *D*_A_ genetic distances [30] between populations were calculated with POPULATIONS 1.2.3 [31] and used to conduct a Principal Coordinates Analysis (PCO) and a Minimum Spanning Tree (MST) that was superimposed on the PCO plots using NTSYSpc 2.11.

Population genetic structure was investigated by means of Analysis of Molecular Variance (AMOVA) which was performed in ARLEQUIN 3.11 to partition in different population groups according to the taxonomical or geographical membership. The significance of the analyses was tested with 1000 replicates.

Bayesian clustering was also used to infer population genetic structure using STRUCTURE 2.1 [32]. Analyses were based on an admixture ancestry model with correlated allele frequencies, for a range of *K* genetic clusters from one to 19, with ten replicates for each *K*. The analyses were performed with a burn-in period and a run length of the Monte Carlo Markov Chain (MCMC) of 7×10^5^ and 7×10^6^ iterations, respectively. The most likely number of genetic clusters (*K*) was determined according to Evanno *et al.* [33].

### Plastid DNA Data Analyses

Haplotype polymorphism was estimated within populations and within genetic and geographical groups through the analysis of the number of segregating sites (*S*), the number of haplotypes (*h*), the haplotype diversity index (*Hd*) and the average number of pairwise nucleotide differences between DNA sequences (θπ) [34] with DnaSP5 [35]. Indels encompassing two to several nucleotides were reduced to single gaps and treated as a fifth nucleotide state for a statistical parsimony haplotype network analysis with TCS v. 1.21 software [36].

### Environmental Niche Modelling Analyses

Environmental niche modelling (ENM) was conducted to evaluate the potential distribution of the geographical groups of *D. humilis* under current climatic conditions and under Last Glacial Maximum (LGM) and Last Interglacial (LIG) conditions. A set of 19 bioclimatic variables (Table S1 in Appendix S1) retrieved from WorldClim (www.worldclim.org) plus the altitude were used, and GIS layers with 30 sec resolution were clipped to the extent of central Chilean regions using DIVA-GIS [37]. Correlation among environmental variables was determined by Mantel tests using XLSTAT and tested for significance with 1000 random permutations (Table S1 in Appendix S1). Then we selected a reduced set of nine uncorrelated environmental variables with higher percent contribution (PC) and permutation importance (PI) based on jackknife pseudosampling on the ENM of *D. humilis* (Tables S1 and S2 in Appendix S1): altitude, bio3 (isothermality), bio4 (temperature seasonality), bio6 (minimum temperature of coldest month), bio7 (annual range temperature), bio9 (mean temperature of driest quarter), bio15 (precipitation seasonality), bio18 (precipitation of warmest quarter) and bio19 (precipitation of coldest quarter).

Additionally, we assessed pairwise correlations between all 20 environmental variables studied and pairwise *D*_A_ population genetic distances [30], and pairwise population linearized *F*_ST_ [28], among populations of *D. humilis*, and the correlation between the 20 environmental variables and latitude, using the Mantel test with 1000 random permutations.

The maximum entropy algorithm implemented in MAXENT v. 3.3.3k [38]–[39] was used to construct the models. Maxent is optimal for ENM when using small sample sizes [40] and when environmental predictions are poorly influenced by the addition of irrelevant bioclimatic variables [41]. The *D. humilis* data fit these requirements since no significant increase in the area under the curve (AUC) values was observed when using all variables compared to those from the reduced set of variables (Table S3 in Appendix S1).

Occurrence data were split into training data (75%) to build the model and test data (25%) to test the accuracy of the model. Fifteen subsample replicates were performed in each run using the default options and 1000 iterations. Model accuracy was assessed with the AUC value of the receiver-operating characteristic curve (ROC) [38]. The contribution of each environmental variable to the ENM was evaluated through a Jackknife pseudosampling (see above). A tenth percentile threshold was applied for all models.

ENM were conducted for the two northern (Dhh01-Dhh08) and southern (Dhh09-Dhh15) population groups of *D. humilis* ssp. *humilis*. The low number of known populations of *D. humilis* subsp. *polyanthes* (Dhp01-02) precluded a confident ENM analysis of this taxon.

ENMs were projected to LGM (c. 21 ka BP), with 2.5 arc-minutes resolution [42], and to LIG (c. 120–140 ka BP), with 30 arc-seconds resolution [43] scenarios. Two palaeoclimatic layers simulated for two general atmospheric circulation models were used for LGM: the Community Climate System Model (CCSM, [44]) and the Model for Interdisciplinary Research on Climate (MIROC, [45]). Both CCSM and MIROC layers were combined following a conservative approach by including their overlapping predicted areas [46]. Current minimum predicted values were used to determine the past minimal predicted areas, assuming that the environmental requirements of *D. humilis* subsp. *humilis* have remained stable during at least since LIG.

A complementary ENM approach was done through a Principal Component Analysis (PCA), which was constructed with the raw data obtained from the 19 climatic variables and the altitude for each population of *D. humilis* using PAST 2.17c [47], Fig. S1 in Appendix S1).

## Results

### Microsatellite Genetic Diversity in *Dioscorea humilis*

All eight microsatellite loci were polymorphic and amplified a total of 79 alleles in the 17 studied populations of *D. humilis* (Table S4 in Appendix S2). The number of alleles per locus ranged from three (B633) to 24 (H442) with a mean of 9.88±6.38 (±SD) alleles per locus. The mean number of alleles per locus and population ranged from 3.25 (Dhh12) to 5.00 (Dhh11, Table 1). Of the 79 microsatellite alleles scored, 34 (43.04%) were shared by both subspecies of *D. humilis*, while 36 (45.57%) and 9 (11.39%) were exclusive to *D. humilis* subsp. *humilis* and *D. humilis* subsp. *polyanthes*, respectively (Table S4 in Appendix S2).

Observed heterozygosities ranged from 0.350 (Dhh04) to 0.598 (Dhp01), and unbiased expected heterozygosities from 0.357 (Dhh12) to 0.521 (Dhh10) (Table 1). Five of the 17 populations showed HW deviations towards heterozygote deficiency; three, including one population of *D. humilis* subsp. *polyanthes*, showed non-significant departure from HW equilibrium, and the remaining eight populations of *D. humilis* subsp. *humilis* and one of *D. humilis* subsp. *polyanthes* showed a significant heterozygote excess (Table 1).

No significant differences were detected for the tested genetic diversity indices between *D. humilis* subsp. *humilis* and *D. humilis* subsp. *polyanthes*, except for observed heterozygosity (*H*_o_). Surprisingly, the more restricted endemic *D. humilis* subsp. *polyanthes* showed significantly higher (*p* = 0.033) average *H*_o_ (Table 1). Similarly, the comparison of northern and southern population groups of *D. humilis* subsp. *humilis* failed to find significant differences at any of the tested indices (Table 1).

### Population Structure of *Dioscorea humilis*

Moderate but significant (different from zero; *p*<0.05) levels of population differentiation were observed among populations (results not shown). Higher average *F*_ST_ values were found between populations of both subspecies (average *F*_ST_ = 0.295) than among populations within subspecies (average Dhh *F*_ST_ = 0.145; Dhp *F*_ST_ = 0.011). Similarly, a higher average differentiation was observed between northern and southern populations groups of *D. humilis* subsp. *humilis* (average *F*_ST_ = 0.198), than among populations within northern populations (Dhh01-Dhh08 *F*_ST_ = 0.069) and southern populations (average Dhh09-Dhh15 *F*_ST_ = 0.109) of *D. humilis* subsp. *humilis* with differentiation between the groups not being statistically significant (Table 2).

**Table 2:** Analyses of molecular variance (AMOVA) of *Dioscorea humilis* populations based on microsatellite data.

Source of variation (groups)	Sum of squared deviations (SSD)	d.f.	Variance components	% of the total variance
**1. ** ***Dioscorea humilis*** ** s.l.**				
Among populations	443.049	16	0.39637	19.03
Within populations	1852.940	1099	1.68602	80.97
**2. Taxonomic membership: ** ***humilis*** ** (Dhh01-Dhh15) ** ***vs*** **. ** ***polyanthes*** ** (Dhp01. Dhp02)**				
Among groups	150.915	1	0.53219	21.37
Among populations within groups	292.314	15	0.27222	10.93
Within populations	1852.940	1099	1.68602	67.70
**3. ** ***Dioscorea humilis*** ** subsp. ** ***humilis s.l.***				
Among populations	288.727	14	0.29176	15.00
Within populations	1589.255	961	1.65375	85.00
**4. Geographical membership of ** ***D. humilis*** ** subsp. ** ***humilis*** **: northern (Dhh01–08), ** ***vs*** **. southern (Dhh09–15)**				
Among groups	133.969	1	0.25056	12.15
Among populations within groups	154.758	13	0.15781	7.65
Within populations	1589.255	961	1.65375	80.20
**5. Genetic membership (excluding ** ***D. humilis*** ** susbp. ** ***polyanthes*** **): cluster Dhh01, Dhh05, Dhh07-Dhh08 ** ***vs*** **. cluster Dhh02-Dhh04, Dhh06 ** ***vs*** **. Dhh09-Dhh10 ** ***vs*** **. Dhh11-Dhh15**				
Among groups	194.783	3	0.23571	11.81
Among populations within groups	93.944	11	0.10659	5.34
Within populations	1589.255	961	1.65375	82.85

Bayesian analysis of population structure showed a maximum Δ*K* = 1598.45 value for *K* = 3 (Fig. S2 in Appendix S2). In this clustering, individuals of *D. humilis* subsp. *polyanthes* showed a high proportion of membership to cluster 3 and those of *D. humilis* subsp. *humilis* to cluster 1 (populations Dhh01-Dhh08) or to cluster 2 (populations Dhh09-Dhh15; Fig. 1a). Mean *F*_ST_ values corresponding to the divergence between clusters 1, 2 and 3 and the hypothetical ancestral population were 0.114, 0.201 and 0.266, respectively, indicating that populations showing a higher membership to cluster 1 were less diverged from the ancestral population. A further maximum Δ*K* = 127.90 value was obtained for *K* = 5 (Fig. S2 in Appendix S2) which separated the populations of *D. humilis* subsp. *humilis* into two additional genetic clusters (clusters 1–4; Fig. 1b).

Non-hierarchical AMOVA attributed 19.03% of the total variation to among populations of *D. humilis s.l*., and 15.00% of the total variation to among populations of *D. humilis* subsp. *humilis* (Table 2). In hierarchical AMOVA, the largest proportion of variation among groups (21.37%) was obtained for a taxonomical grouping of populations into subspecies. AMOVA based on a geographical grouping of populations attributed 12.15% of the variation to differences between northern and southern groups of *D. humilis* subsp. *humilis* and a lower proportion of variance (7.65%) to differences among populations within groups (Table 3). The grouping of *D. humilis* subsp. *humilis* populations into four genetic clusters did not increase the variance among groups (11.81%) but lowered the proportion of variance among populations within groups (5.34%).

**Table 3:** Plastid combined *trn*TL- *trn*LF haplotype diversity analysis of *D. humilis* populations and geographical/genetic groups.

Population/Group	*N*	*S*	*h*	*Hd*	θπ
***D. humilis ssp humilis*** ** (**Dhh01-15**)**	45	6	6	0.391	0.789 (0.000–2.404)
**Northern range** (Dhh01-08)	27	3	3	0.325	0.873 (0.000–2.610)
Dhh01-06	21	3	3	0.400	1.089 (0.000–3.124)
Dhh07-08	6	0	1	0.000	-
**Dhh01**	5	1	2	0.400	0.397 (0.000–1.800)
**Dhh02**	2	0	1	0.000	-
**Dhh03**	2	0	1	0.000	-
**Dhh04**	4	0	1	0.000	-
**Dhh05**	4	0	1	0.000	-
**Dhh06**	4	0	1	0.000	-
**Dhh07**	3	0	1	0.000	-
**Dhh08**	3	0	1	0.000	-
**Southern range** (Dhh09-15)	18	3	4	0.477	0.534 (0.000–1.863)
Dhh09-10	4	1	2	0.667	0.664 (0.000–2.500)
Dhh11-15	14	2	3	0.385	0.406 (0.000–1.560)
**Dhh09**	2	0	1	0.000	-
**Dhh10**	2	0	1	0.000	-
**Dhh11**	2	0	1	0.000	-
**Dhh12**	2	0	1	0.000	-
**Dhh13**	3	0	1	0.000	-
**Dhh14**	4	1	2	0.667	0.673 (0.000–2.500)
**Dhh15**	3	1	2	0.667	0.656 (0.000–2.667)
***D. humilis ssp polyanthes*** ** (**Dhp01-02**)**	9	1	2	0.500	0.506 (0.000–1.889)
**Dhp01**	3	0	1	0.000	-
**Dhp02**	6	0	1	0.000	-
**Total**	54	7	7	0.419	0.768 (0.000–2.379)

Population codes, sample size (*N*), and combined *trn*TL- *trn*LF haplotype frequency parameters: number of segregating sites (*S*), number of distinct haplotypes (*h*), and haplotype diversity (*Hd*) and molecular diversity (θπ) estimates (with 95% confidence intervals of θπ generated through 10,000 θ-based simulations under the coalescence model using the program DNAsp v.5 [35].

**Figure 2:**
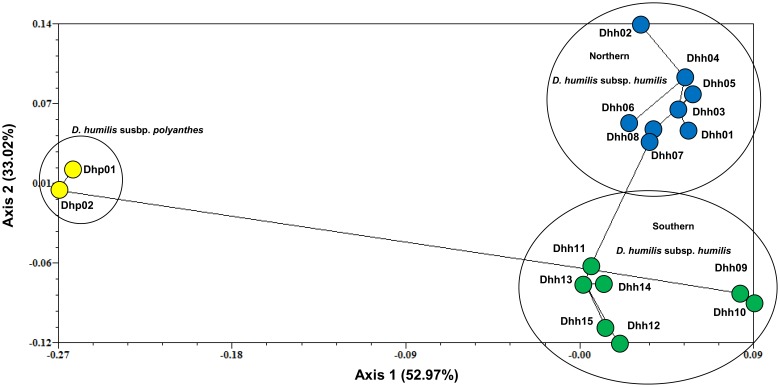
Principal Coordinates Analysis (PCO) showing the genetic relationships among populations of *Dioscorea humilis* based on *D*_A_ genetic distance [30]. Populations of *D. humilis* subsp. *polyanthes* (Dhp01, Dhp02), yellow circles; northern populations of *Dioscorea humilis* subsp. *humilis* (Dhh01 to Dhh08), blue circles; southern populations of *D. humilis* subsp. *humilis* (Dhh09 to Dhh15), green circles.

PCO showed results consistent with STRUCTURE (Figs. 1 and 2). Populations of *D. humilis* subsp. *polyanthes* separated at a large distance from populations of *D. humilis* subsp. *humilis* (Fig. 2). Clustering of populations of this latter taxon was consistent with their geographical distribution (Fig. 2). PCO with superimposed MST analysis identified the closer relationship of *D. humilis* subsp. *polyanthes* to the southern populations of *D. humilis* subsp. *humilis* (Fig. 2).

**Figure 3:**
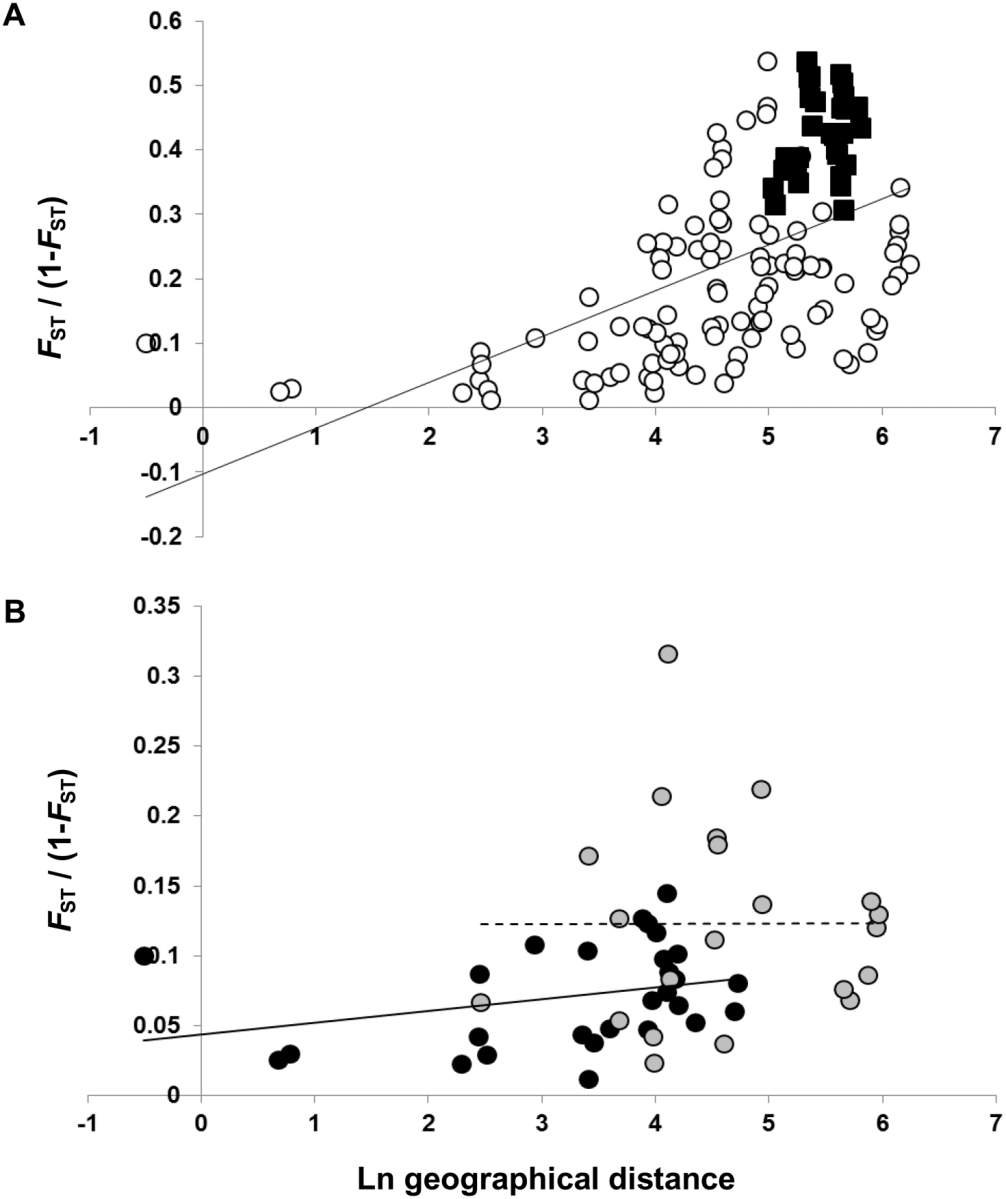
Isolation by distance analyses. Correlation between log-transformed pairwise geographical distances and linearized *F*_ST_ values [28] among populations of *D. humilis*. A. *D. humilis s.l.* where open circles represent pairwise comparisons among populations of *D. humilis*. subsp. *humilis* and black squares represent pairwise comparisons among populations of *D. humilis*. subsp. *humilis* and *D. humilis* subsp. *polyanthes*. Correlation between matrices was *r* = 53.74%, *p* = 0.001 for *D. humilis s.l*. and *r* = 41.61%, *p* = 0.004 for *D. humilis* subsp. *humilis* B. IBD analyses within geographical groups of *D. humilis* susbp. *humilis*, where black circles represent pairwise comparisons among populations of the northern group (Dhh01-Dhh08) and grey circles represent pairwise comparisons among populations of the southern group (Dhh09-Dhh15). Correlation between matrices was *r* = 29.62%, *p* = 0.080 and *r* = 0.47%, *p* = 0.420 for the northern and southern groups, respectively.

A significant correlation between pairwise geographical distances and linearized *F*_ST_ values was found in both *D. humilis s.l*. (*r* = 0.537, *p* = 0.001) and *D. humilis* subsp. *humilis* (*r* = 0.416, *p* = 0.004) populations (Fig. 3a), thus showing significant isolation by distance (IBD). However, the pattern of IBD vanished when this analysis was separately conducted within both northern (*r* = 0.296, *p* = 0.080) and southern (*r* = 0.005, *p* = 0.420) geographical groups of *D. humilis* subp. *humilis* (Fig. 3b).

### Plastid Haplotype Diversity in *Dioscorea humilis*

**Figure 4:**
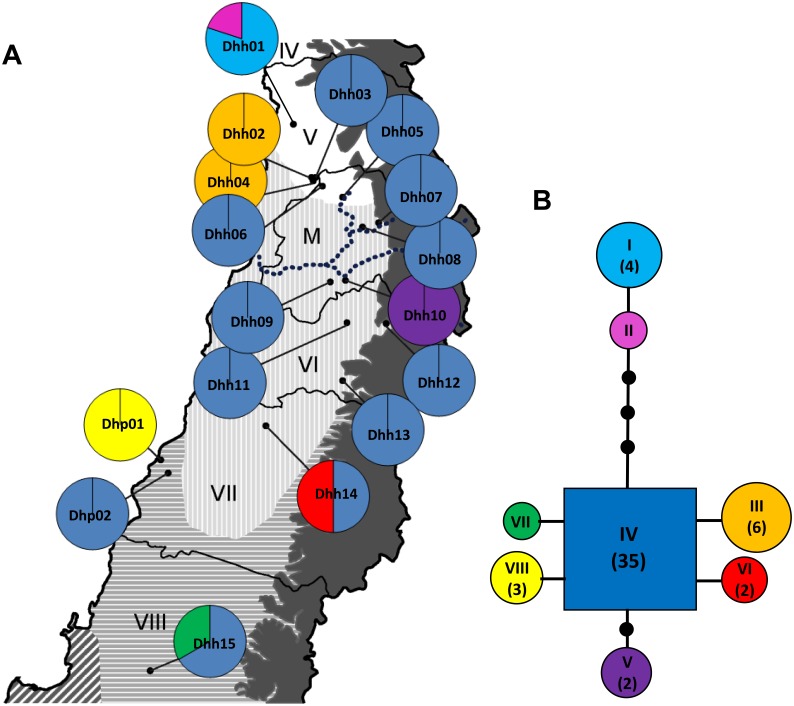
Plastid haplotype diversity in *Dioscorea humilis*. A. Geographical distribution of eight chloroplast haplotypes in 17 populations of *Dioscorea humilis*. Pie charts indicate relative frequencies of each haplotype in each population. The dotted line indicates the location of the Maipo river. Chilean administrative regions and climatic regions are indicated as in Fig. 1. B. Parsimony Network showing the relationships among eight haplotypes. Black dots indicate unsampled or extinct haplotypes. The size of the circles or squares is proportional to the number of sequences representing each haplotype, and is indicated in parentheses when higher than one. Map contour constructed from spatial data retrieved from http://www.diva-gis.org/gdata.

The combination of *trn*L–F and *trn*T–L plastid DNA regions produced eight haplotypes (Fig. 4; Table S5 in Appendix S2). Six and one haplotypes were restricted to *D. humilis* subsp. *humilis* and subsp. *polyanthes* respectively, and one haplotype was shared between both taxa. Haplotype IV was widespread in 12 populations of *D. humilis*, including one population of *D. humilis* subsp. *polyanthes*, and had the highest outgroup probability (0.771). Three haplotypes (I, II and III) were restricted to some western populations of northern *D. humilis* subsp. *humilis*, whereas haplotypes V, VI and VII were restricted to some southern populations of *D. humilis* subsp. *humilis* (Fig. 4a; Table S5 in Appendix S2).

Haplotype diversity was higher in *Dioscorea humilis* subsp. *humilis* (*S* = 6, θπ = 0.789), than in *D. humilis* subsp. *polyanthes* (*S* = 1, θπ = 0.500) (Table 3), as expected for the wider distribution range and population abundance of the former. The northern group of populations of *D. humilis* subsp. *humilis* (Dhh01-Dhh08) showed higher haplotype diversity (*S* = 3, θπ = 0.873) compared to the southern group (Dhh09-Dhh15) of populations (*S* = 3, θπ = 0.534) (Table 3).

TCS estimated a 95% maximum connection of 17 steps incorporating all eight haplotypes into the network and inferred three missing haplotypes (Fig. 4b). The haplotype network showed a star-like pattern with four of the six derived haplotypes directly connected to the most widespread one (Hap. IV; Fig. 4). Three haplotypes were connected to the central one at a larger number of mutations. Two of them were private to the northernmost population (Hap. I and II) whereas the other (Hap. V) was private to a population from the southern group (Fig. 4).

### Environmental Niche Modelling

All rainfall-derived variables (bio12-bio19) and all but three temperature-derived variables (bio6, bio8 and bio11) were significantly correlated to genetic distances (Table S1 in Appendix S1). Also, latitude was highly correlated to all rainfall-derived variables (bio12-bio19), to two temperature-derived variables (bio2-bio3), and to both pairwise populations’ *D*_A_ and *F*_ST_ genetic distances (Table S1 in Appendix S1).

**Figure 5:**
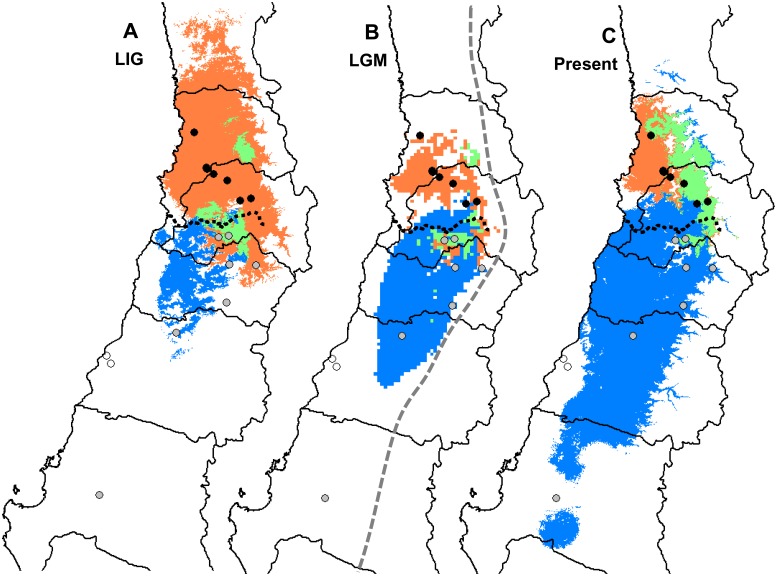
Environmental Niche Modelling (ENM) of *Dioscorea humilis* estimated under Last Interglacial (LIG) (A), Last Glacial Maximum (LGM) (B) and current (C) climate conditions. In orange, northern *D. humilis* subsp. *humilis* genetic group, in blue, southern genetic group and in bright green, overlapping areas among predicted distributions. A tenth percentile threshold was applied. Black circles: northern populations of *D. humilis* subsp. *humilis* (Dhh01 to Dhh08); grey circles: southern populations of *D. humilis* subsp. *humilis* (Dhh09 to Dhh15). The black dashed line indicates the location of the Maipo river. The grey dashed line in B indicates the approximate extent of the ice sheet during the LGM after [5]. Map contours constructed from spatial data retrieved from http://www.diva-gis.org/gdata.

According to response curves and Jackknife tests, the most informative variables for the ENM of *D. humilis s.l*. were altitude and three climatic variables derived from rainfall data (bio16, precipitation wettest quarter; bio18, precipitation warmest quarter; and bio19, precipitation coldest quarter). At the subspecies level, the variables with the largest contributions to the ENM of *D. humilis* subsp. *humilis* were altitude and the climatic variables bio8 (mean temperature of wettest quarter), bio9 (mean temperature of driest quarter) and bio15 (precipitation seasonality) (Table S2 in Appendix S1). Independent ENM for northern (Dhh01-Dhh08) and southern (Dhh09-Dhh15) genetic groups of *D. humilis* subsp. *humilis* revealed that the variables bio15 and bio18 were most informative for the northern group, whereas bio8, bio9 and bio15 were most informative for the southern group (Table S2 in Appendix S1). All projections (Figs. 5a–5c) showed excellent predictive success rates, with AUC values higher than 0.9 (Table S3 in Appendix S1). The PCA of environmental variables for the *D. humilis* populations (Fig. S1 in Appendix S1) separated northern from southern genetic groups of *D. humilis* subsp. *humilis*. The southernmost population, Dhh15, showed a distinct set of climatic conditions from the others, according to its separated position, and clustered together with the *D. humilis* subsp. *polyanthes* populations in the bidimensional PCA plot (Fig. S1 in Appendix S1).

The ENM for current environmental conditions was mostly concordant with the current distribution of northern and southern genetic groups of *D. humilis* subsp. *humilis* (Fig. 5c), except for the southernmost population, Dhh15. Potential areas of contact were predicted on the eastern boundary of their distributions. Projections to the LIG (Fig. 5a) predicted a minimal extension area for the potential distribution of the southern group and a larger extension for the northern group. Projections to the LGM (Fig. 5b) predicted a substantial reduction in the southern group and, to a lesser extent, of the northern group. The present and the two historical models predicted areas with unsuitable environmental conditions between the northern and southern groups (Fig. 5), and an increase in the potential areas of contact between the two groups since the LGM to the present.

## Discussion

### Genetic Diversity, Genetic Structure and Diversification of *Dioscorea humilis*

Genetic diversity and population structure in plant species is determined by various abiotic and biotic factors, some of which have triggered population differentiation [48] and speciation processes [49]–[50]. Biotic factors have been globally assigned to life-history (e.g. life form), and reproductive traits (e.g. reproductive systems, pollination and seed dispersal mechanisms [51]). However, closely related taxa, such as the two subspecies of *D. humilis*, show similar biotic parameters. Abiotic factors include, as most relevant, climatic variables and barriers to dispersal. Our results suggest that the conjunction of these two later factors caused the intraspecific split within *D. humilis*.

Our analyses revealed moderate levels of allelic diversity and heterozygosity across populations of *D. humilis* (*A* = 3.25–5.0, *H*_O_ = 0.350–0.598, *H*_E_ = 0.357–521, Table 1), which were relatively lower than in the sister species *D. biloba* (*A* = 5.14–7.29, *H*_O_ = 0.345–0.686, *H*_E_ = 0.458–0.706, Table S6 in Appendix S3). However, genetic diversity parameters of *D. humilis* were in the range of other yam species with a different combination of life-history (climbers), reproductive (winged seeds) and distribution range (broad range, non-endemic), characteristics that, contrary to *D. humilis* should predispose them to higher levels of genetic diversity (Table S6 in Appendix S3). By contrast, *D. humilis* showed higher genetic diversity than those of species of the *Borderea* group of *Dioscorea* which are comparable in morphological and reproductive traits (Table S6 in Appendix S3), though the Pyrenean *Dioscorea* species differ from *D. humilis* in their even narrower distributions [52]–[53].

Widespread taxa tend to maintain higher levels of genetic diversity compared to geographically restricted congeners [54]. However, genetic diversity in *D. humilis* subsp. *polyanthes* was not significantly lower than in *D. humilis* subsp. *humilis*, despite its more restricted geographical range (Table 1). This result could suggest equally or more efficient mechanisms buffering against genetic loss in *D. humilis* subsp. *polyanthes*.

Our study also revealed a strong geographical structure of nuclear microsatellite variation throughout the range of *D. humilis* (Fig. 1), with populations of *D. humilis* subsp. *polyanthes* clearly separate from those of *D. humilis* subsp. *humilis* that split into clusters 1 (northern populations Dhh01-Dhh08) and 2 (southern populations Dhh09-Dhh15; Fig. 1a). Clustering analyses (Fig. 2) and AMOVA (Table 3) also found a major differentiation between the two subspecies, in agreement with their morphological distinction [16]. However, plastid DNA haplotype sharing (haplotype IV) between the subspecies and the occurrence of a private plastid haplotype (VIII) in Dhp01, directly derived from the most common haplotype (IV), suggests recent diversification mediated by isolation by distance (Fig. 3a), with incomplete lineage sorting in *D. humilis* subsp. *polyanthes* (Fig. 4) or alternatively, introgression between subspecies.

Bayesian *F*_ST_ values supported an origin of the species in the northern region and a derived recent origin of *D. humilis* subsp. *polyanthes* from the southern group of *D. humilis* subsp. *humilis*. This was corroborated by the highest haplotypic diversity of the northern group (Table 3) and by the PCO-MST analysis, which indicated the closeness of *D. humilis* subsp. *polyanthes* to the southern *D. humilis* subsp. *humilis* populations (Fig. 2).

The intraspecific divergence of the southern *D. humilis* subsp. *polyanthes* from the southern group of *D. humilis* subsp. *humilis* could have been a consequence of local environmental adaptation. However, despite the lack of an ENM for the former taxon, the range of values for its 19 bioclimatic variables overlap with that of the latter group and are not significantly different from them (Table S2 in Appendix S1). Thus, the explanation for their divergence is other than a climatically driven speciation process; it may be rather the consequence of geographical isolation and incomplete plastid lineage sorting of *D. humilis* subsp. *polyanthes* from the central Chilean depression southern *D. humilis* subsp. *humilis* group during the last glacial and interglacial phases (Fig. 5).

### Influence of Current and Past Latitudinal Climatic Heterogeneity on the Genetic Structure of *Dioscorea humilis* subsp. *humilis*

A noticeable finding was the strong geographical structure detected among *D. humilis* subsp. *humilis* populations, which separated into two North-to-South genetic groups (Fig. 1a). Such spatial patterns are usually driven by the effect of strong geographical or climatic barriers to dispersal, as proposed for *Hordeum chilense* Roem. & Schult., which is similarly distributed along a climatic gradient in Chile [20]. The distribution of the genetic groups of *D. humilis* subsp. *humilis* mostly paralleled those of the main Chilean latitudinal climatic zones (Fig. 1). Mountain chains in the central Chilean depression show lower altitudes and may not significantly contribute to latitudinal isolation of populations. Our study showed that the genetic divergence of the two population groups occurred northwards and southwards of the Maipo river basin (Fig. 1). Indeed, river basins have been identified as efficient barriers to dispersal for seed plants, such as in Chinese populations of *Vitex negundo* L. (Verbenaceae) on opposite shores of the Yangtze river [55]. Specifically, the role of the Maipo river as a geographical barrier has been highlighted for other organisms with potentially higher dispersal capabilities than *D. humilis*, such as the snake *Philodryas chamissonis* Wiegmann [14]. The Maipo river acting as a geographical barrier to gene flow could contribute to explain the IBD pattern across the range of *D. humilis* subsp. *humilis* (Fig. 3a), and the abrupt difference in genetic structure between the two geographical groups (Fig. 1a). However, this IBD pattern does not apply for within-group pairwise population comparisons (Fig. 3b). The absence of IBD within the two geographical areas of *D. humilis* subsp. *humilis* contrasts with life-history and reproductive traits of the species which all point towards extremely short dispersal distances [16]. Therefore, the observed patterns are probably mirroring historical gene flow among populations within ranges preceding range expansions in the Holocene and a relatively rapid postglacial expansion by unknown vectors.

Geographical and historical variations of environmental variables have been demonstrated to greatly influence genetic divergence among populations [20], [56]. Our ENM analyses indicate a strong latitudinal ecological differentiation throughout the current range of *D. humilis* subsp. *humilis* into two well defined environmental niches (Fig. 5c). Past projection of niche models indicate that this ecological niche differentiation likely originated earlier than LIG (Fig. 5a), and that ecological conditions have been maintained until present times. The current separation of the groups by the Maipo river basin [14] matches the ecogeographical division of the *D. humilis* range into northern semiarid and southern subhumid Mediterranean areas (Fig. 1, [20]). Ice-cover during the LGM, which reached to 35°S in the Andes [5] [6]), together with its northwards influence that extended to approximately 33°S, could have strengthened the barrier effect of the Maipo river. Water volume of this river originating from the Andes was likely higher during LGM than in present times [14], which could account for the allopatric distribution of the two population groups, thereby contributing to the observed genetic differentiation between them (Fig. 5). Bayesian *F*_ST_ values supported the ancestry of the northern populations, suggesting a likely origin of the species in its northern range, in the overlapping area with its congener *D. biloba* [16], followed by further southwards expansion.

Nonetheless, the predicted extension of the potential distribution areas of the two population groups could have fluctuated both in latitude and longitude during glacial and interglacial episodes, as expected from changes in climatic parameters in those areas following periods of warming (LIG and present) and cooling (LGM, Fig. 5). The predicted distribution area of the northern population group showed a maximum extension during LIG (Fig. 5a), whereas the strong reduction during LGM (Fig. 5b) was maintained until present times (Fig. 5c). Contrastingly, the predicted distribution area of the southern group showed a progressive increase in extension from LIG (Fig. 5a) to present times (Fig. 5c). The potential overlap of distribution areas between the two groups reached its maximum extension during present times (Fig. 5c). However, it was restricted to the eastern range of the present distribution of the species, suggesting that lineage migration and admixture, as denoted by the occurrence of the common plastid haplotype IV, is likely to have occurred only along the eastern boundaries of both distribution areas (Fig. 5). Predicted environmental niche models of the northern and southern groups of *D. humilis* were also consistent with a contraction towards the central Chilean depression during the LGM (Fig. 5b), preceded by broader eastern and western distributions of the potential areas of the northern group during the LIG (Fig. 5a).

Bayesian analyses of nuclear microsatellite variation and of plastid haplotypes also revealed genetic signatures of postglacial population expansion within the northern and southern groups of *D. humilis* subsp. *humilis* (Figs. 1b, 4a). Concerning the northern group, western populations predominantly showed a microsatellite genetic membership to cluster 1, whereas eastern populations showed a predominant genetic membership to cluster 2 (Fig. 1a). This was paralleled to a lesser extent by the slower-evolving cpDNA data (Fig. 4a), where three northwestern populations showed three cpDNA haplotypes that were not represented in eastern populations (Fig. 4a). This would indicate a further isolation of the northwestern populations, which, unlike the eastern ones, did not admix with the southern ones.

Contrastingly, a North-to-South expansion was detected in the southern group of *D. humilis* subsp. *humilis*, supported by a gradual North-to-South decrease in microsatellite genetic membership to cluster 4, and an increase in membership to cluster 3 (Fig. 1b), agreeing with the predicted postglacial southwards expansion (Fig. 5c). Exclusive cpDNA haplotypes were scattered among populations in this range, and were all derived directly from the most common haplotype (Fig. 4b), suggesting recent divergence and dispersal [57]–[58].

## Conclusions

Our study represents a significant contribution to the understanding of the phylogeography of lowland plants from the central Mediterranean area of Chile. Genetic and ENM analyses suggest that *D. humilis* subsp. *polyanthes* diverged from southern populations of *D. humilis* subsp. *humilis* due to local niche adaptation to coastal areas.

The study has also revealed a strong phylogeographical structure within *D. humilis* subsp. *humilis* and identified two highly differentiated genetic groups with distributions that match present latitudinal environmental heterogeneity in the area [20], [59]. The genetic differentiation of these two groups could have been triggered by a coupled effect of adaptation to divergent ecological parameters of higher and lower aridity in the northern and southern geographical areas, respectively [60]–[62], enhanced by the permanent geographical barrier of the Maipo river basin between the two areas [14].

## Supporting Information

Appendix S1 **Environmental niche model analysis of** ***Dioscorea humilis*** **.**
(DOC)

Appendix S2 **Microsatellite allele frequencies, Bayesian estimation of genetic clusters and plastid haplotype frequencies in populations of** ***Dioscorea humilis*** **.**
(DOC)

Appendix S3 **Comparison of microsatellite genetic diversity in yam species.**
(DOC)
