# Supplementary material for: Latitudinal Environmental Niches and Riverine Barriers Shaped the Phylogeography of the Central Chilean Endemic Dioscorea humilis (Dioscoreaceae)
Source: PLoS One. 2014 Oct 8;9(10):e110029. doi: 10.1371/journal.pone.0110029 (PMC4190404; doi:10.1371/journal.pone.0110029)
Supplement: Appendix S1 — Environmental niche model analysis of Dioscorea humilis . (DOC) [file pone.0110029.s001.doc]

| **Table S1.**  Bioclimatic variables used in the environmental niche modeling (ENM) of the *Dioscorea humilis* s. l. taxonomic and genetic groups. Pairwise Mantel correlations between the 19 WorldClim variables, the altitude, two genetic indices and latitude of *Dioscorea humilis.* Significance of Mantel tests are based on 10000 permutations. | | | | | | | | | | | | | | | | | | | | | |
| --- | --- | --- | --- | --- | --- | --- | --- | --- | --- | --- | --- | --- | --- | --- | --- | --- | --- | --- | --- | --- | --- |
|  | Altitude | Temperature | | | | | | | | | | | Rainfall | | | | | | | Latitude | |
| Variables | **alt** | bio1 | bio2 | **bio3** | **bio4** | bio5 | **bio6** | **bio7** | bio8 | **bio9** | bio10 | bio11 | bio12 | bio13 | bio14 | **bio15** | bio16 | bio17 | **bio18** | **bio19** | Lat |
| Altitude | - |  |  |  |  |  |  |  |  |  |  |  |  |  |  |  |  |  |  |  |  |
| Annual Mean Temperature | 0.000^ns^ | - |  |  |  |  |  |  |  |  |  |  |  |  |  |  |  |  |  |  |  |
| Mean Diurnal Range (Mean of monthly (max temp - min temp)) | **0.622***** | 0.035^ns^ | - |  |  |  |  |  |  |  |  |  |  |  |  |  |  |  |  |  |  |
| Isothermality (BIO2/BIO7) (* 100) | -0.109^ns^ | -0.117^ns^ | -0.055^ns^ | - |  |  |  |  |  |  |  |  |  |  |  |  |  |  |  |  |  |
| Temperature Seasonality (standard deviation *100) | 0.170^ns^ | -0.004^ns^ | 0.306* | **0.533***** | - |  |  |  |  |  |  |  |  |  |  |  |  |  |  |  |  |
| Max Temperature of Warmest Month | -0.065^ns^ | 0.277* | 0.252* | 0.200* | **0.600***** | - |  |  |  |  |  |  |  |  |  |  |  |  |  |  |  |
| Min Temperature of Coldest Month | **0.525***** | **0.516***** | 0.262* | -0.109^ns^ | 0.108^ns^ | -0.184* | - |  |  |  |  |  |  |  |  |  |  |  |  |  |  |
| Temperature Annual Range (bio5-bio6) | **0.315***** | 0.171^ns^ | **0.499***** | 0.172* | **0.830***** | **0.624***** | 0.309* | - |  |  |  |  |  |  |  |  |  |  |  |  |  |
| Mean Temperature of Wettest Quarter | 0.004* | **0.656***** | 0.102^ns^ | -0.085^ns^ | 0.094^ns^ | -0.103^ns^ | **0.830***** | 0.272* | - |  |  |  |  |  |  |  |  |  |  |  |  |
| Mean Temperature of Driest Quarter | -0.092^ns^ | **0.581***** | 0.091^ns^ | 0.115^ns^ | 0.268* | **0.772***** | -0.008^ns^ | 0.302* | 0.118^ns^ | - |  |  |  |  |  |  |  |  |  |  |  |
| Mean Temperature of Warmest Quarter | -0.024^ns^ | **0.532***** | 0.239^ns^ | 0.163* | **0.448***** | **0.813***** | -0.004^ns^ | **0.388***** | 0.082^ns^ | **0.858***** | - |  |  |  |  |  |  |  |  |  |  |
| Mean Temperature of Coldest Quarter | 0.185* | **0.707***** | 0.016^ns^ | 0.010^ns^ | 0.099^ns^ | -0.119^ns^ | **0.840***** | 0.224* | **0.874***** | 0.088^ns^ | 0.080^ns^ | - |  |  |  |  |  |  |  |  |  |
| Annual Precipitation | **0.707***** | 0.162^ns^ | 0.263* | 0.074^ns^ | 0.013^ns^ | 0.105^ns^ | -0.073^ns^ | 0.058^ns^ | -0.064^ns^ | 0.036^ns^ | 0.102^ns^ | 0.032^ns^ | - |  |  |  |  |  |  |  |  |
| Precipitation of Wettest Month | -0.015^ns^ | 0.242* | 0.113^ns^ | 0.201* | -0.018^ns^ | 0.094^ns^ | -0.051^ns^ | 0.019^ns^ | 0.005^ns^ | 0.080^ns^ | 0.044^ns^ | 0.139^ns^ | **0.812***** | - |  |  |  |  |  |  |  |
| Precipitation of Driest Month | 0.065^ns^ | 0.116^ns^ | 0.155^ns^ | 0.029^ns^ | -0.036^ns^ | 0.075^ns^ | -0.119^ns^ | 0.006^ns^ | -0.093^ns^ | 0.018^ns^ | 0.051^ns^ | -0.001^ns^ | **0.916***** | **0.779***** | - |  |  |  |  |  |  |
| Precipitation Seasonality (Coefficient of Variation) | -0.024^ns^ | 0.127^ns^ | 0.010^ns^ | 0.126^ns^ | -0.029^ns^ | 0.037^ns^ | -0.053^ns^ | -0.024^ns^ | -0.025^ns^ | -0.036^ns^ | 0.028^ns^ | 0.053^ns^ | **0.587***** | **0.518***** | **0.688***** | - |  |  |  |  |  |
| Precipitation of Wettest Quarter | 0.094^ns^ | 0.177* | 0.266* | 0.112^ns^ | 0.007^ns^ | 0.097^ns^ | -0.058^ns^ | 0.046^ns^ | -0.050^ns^ | 0.042^ns^ | 0.097^ns^ | 0.053^ns^ | **0.984***** | **0.835***** | **0.859***** | **0.493***** | - |  |  |  |  |
| Precipitation of Driest Quarter | 0.030^ns^ | 0.105^ns^ | 0.068^ns^ | 0.021^ns^ | -0.063^ns^ | 0.014^ns^ | -0.075^ns^ | -0.010^ns^ | -0.055^ns^ | -0.026^ns^ | -0.026^ns^ | 0.019^ns^ | **0.768***** | **0.720***** | **0.916***** | **0.799***** | **0.681***** | - |  |  |  |
| Precipitation of Warmest Quarter | 0.136^ns^ | 0.099^ns^ | 0.241* | 0.001^ns^ | 0.015^ns^ | 0.105^ns^ | -0.086^ns^ | 0.075^ns^ | -0.092^ns^ | 0.020^ns^ | 0.091^ns^ | -0.036^ns^ | **0.906***** | **0.673***** | **0.950***** | **0.727***** | **0.828***** | **0.916***** | - |  |  |
| Precipitation of Coldest Quarter | -0.009^ns^ | 0.267* | 0.151^ns^ | 0.114^ns^ | -0.028^ns^ | 0.130^ns^ | -0.057^ns^ | 0.054^ns^ | -0.004^ns^ | 0.103^ns^ | 0.072^ns^ | 0.117^ns^ | **0.843***** | **0.982***** | **0.815***** | **0.579***** | **0.848***** | **0.778***** | **0.744***** | - |  |
| Latitude | 0.121^ns^ | 0.128^ns^ | **0.207*** | **0.234**** | 0.077^ns^ | 0.084^ns^ | 0.008^ns^ | 0.121^ns^ | 0.013^ns^ | 0.021^ns^ | 0.016^ns^ | 0.085^ns^ | **0.722***** | **0.784***** | **0.729***** | **0.658***** | **0.688***** | **0.759***** | **0.740***** | **0.806***** |  |
| Linearized *F*_ST_ (Slatkin, 1995) | 0.014^ns^ | **0.275**** | **0.227*** | **0.265**** | **0.313**** | **0.541***** | -0.033^ns^ | **0.372***** | -0.012^ns^ | **0.411***** | **0.377****** | 0.082^ns^ | **0.320***** | **0.465***** | **0.276**** | **0.181*** | **0.338**** | **0.238**** | **0.251**** | **0.481***** | **0.333***** |
| *D*_A_ genetic index (Nei *et al*.,1983) | -0.025^ns^ | **0.197**** | **0.171*** | **0.427***** | **0.339***** | **0.494***** | -0.097^ns^ | **0.307***** | -0.072^ns^ | **0.332***** | **0.364***** | 0.027^ns^ | **0.431***** | **0.544***** | **0.399***** | **0.380***** | **0.438***** | **0.372***** | **0.377***** | **0.540***** | **0.310***** |
| Significance: *, p<0.05, **, p<0.01, ***, p<0.0001, ns, not significant. | | | | | | | | | | | | | | | | | | | | | |

| **Table S2.** Contribution and importance of the bioclimatic and altitude variables used in Environmental Niche Modelling analyses performed with Maxent for present, LGM (under MIROC and CCSM models) and LIG conditions. PC = percent contribution, PI = permutation importance, AV = all 20 variables, RV = reduced variable set. | | | | | | | | | | | | | | | | |
| --- | --- | --- | --- | --- | --- | --- | --- | --- | --- | --- | --- | --- | --- | --- | --- | --- |
| **Population groups** | **Dhh01-15, Dhp01,02** | | | | **Dhh01-15** | | | | **Dhh01-08** | | | | **Dhh09-15** | | | |
| **Variable set** | **AV** | | **RV** | | **AV** | | **RV** | | **AV** | | **RV** | | **AV** | | **RV** | |
| **Present** | **PC** | **PI** | **PC** | **PI** | **PC** | **PI** | **PC** | **PI** | **PC** | **PI** | **PC** | **PI** | **PC** | **PI** | **PC** | **PI** |
| **Alt** | **10.3** | **49.2** | **10.3** | **45.5** | **6.7** | **37** | **5.1** | **17.2** | 6.8 | 4.1 | 6.2 | 2.2 | 0.0 | 0.0 | 0.0 | 0.0 |
| **Bio1** | 0.1 | 0.0 | - | - | 0.0 | 0.0 | - | - | 0.1 | 0.0 | - | - | 0.0 | 0.0 | - | - |
| **Bio2** | 0.5 | 1.2 | - | - | 9.1 | 3.4 | - | - | 9.6 | 6.0 | - | - | 0.5 | 0.0 | - | - |
| **Bio3** | 0.8 | 0.5 | 0.5 | 1.2 | 0.9 | 0.0 | 2.1 | 2.5 | 5.4 | 1.5 | 8.5 | 6 | 2.5 | 2.8 | 1.8 | 0.5 |
| **Bio4** | 0.0 | 0.0 | 0.1 | 0.0 | 0.0 | 0.0 | 0.0 | 0.0 | 0.0 | 0.0 | 0.0 | 0.0 | 0.3 | 3.6 | 0.9 | 3.8 |
| **Bio5** | 0.0 | 0.0 | - | - | 0.6 | 0.0 | - | - | 1.2 | 0.4 | - | - | 0.5 | 9.1 | - | - |
| **Bio6** | 3.0 | 3.9 | 3.2 | 1.1 | 1.4 | 0.0 | 4.2 | 3.4 | 0.0 | 0.0 | 0.1 | 0.6 | 0.0 | 0.0 | 0.0 | 0.0 |
| **Bio7** | 0.0 | 0.0 | 0.0 | 0.0 | 0.0 | 0.0 | 4.6 | 4.4 | 0.0 | 0.0 | 4.8 | 5.6 | 2.3 | 8.2 | 3.8 | 1.2 |
| **Bio8** | 0.0 | 0.1 | **-** | **-** | **1.7** | **20.5** | - | - | 0.1 | 1.3 | **-** | **-** | **1.3** | **20.8** | - | - |
| **Bio9** | 0.9 | 0.0 | 1.2 | 0.0 | **42.1** | **0.0** | **45.5** | **1.0** | 0.0 | 0.0 | 7.0 | 0.0 | **69.7** | **25.7** | **70.2** | **64.8** |
| **Bio10** | 0.0 | 0.0 | - | - | 0.0 | 0.0 | - | - | 0.0 | 0.0 | - | - | 0.0 | 0.0 | - | - |
| **Bio11** | 0.0 | 0.0 | - | - | 0.0 | 0.0 | - | - | 0.1 | 0.0 | - | - | 0.0 | 0.0 | - | - |
| **Bio12** | 0.0 | 0.0 | - | - | 0.0 | 0.0 | - | - | 0.0 | 0.0 | - | - | 0.0 | 0.0 | - | - |
| **Bio13** | 0.0 | 0.0 | - | - | 0.0 | 0.1 | - | - | 0.0 | 0.0 | - | - | 2.9 | 0.0 | - | - |
| **Bio14** | 5.0 | 2.6 | - | - | 1.3 | 0.9 | - | - | 7.2 | 1.1 | - | - | 0.0 | 0.0 | - | - |
| **Bio15** | 2.1 | 0.2 | 3.0 | 1.5 | **22.2** | **34.3** | **23.0** | **29.0** | **15.2** | **0.0** | **15.7** | **0.0** | **16.6** | **20.5** | **16.8** | **27.3** |
| **Bio16** | **1.4** | **18.2** | - | - | 0.0 | 0.0 | - | - | 0.0 | 0.0 | - | - | 0.3 | 0.5 | - | - |
| **Bio17** | 2.5 | 1.4 | - | - | 0.4 | 3.2 | - | - | 0.4 | 0.0 | - | - | 0.0 | 0.0 | **-** | **-** |
| **Bio18** | **30.5** | **21.9** | **36.9** | **33.6** | 1.8 | 0.6 | **13.9** | **42.4** | **43.5** | **80.1** | **53.3** | **80.0** | 2.2 | 4.9 | 2.3 | 1.9 |
| **Bio19** | **43** | **0.6** | **44.5** | **16.9** | 1.7 | 0.1 | 1.4 | 0.0 | 4.1 | 5.3 | 4.5 | 5.6 | 0.9 | 3.8 | 4.3 | 0.4 |
|  |  |  |  |  |  |  |  |  |  |  |  |  |  |  |  |  |
| **Population groups** | **Dhh01-15, Dhp01,02** | | | | **Dhh01-15** | | | | **Dhh01-08** | | | | **Dhh09-15** | | | |
| **Variable set** | **AV** | | **RV** | | **AV** | | **VR** | | **AV** | | **RV** | | **AV** | | **RV** | |
| **LGM MICROC** | **PC** | **PI** | **PC** | **PI** | **PC** | **PI** | **PC** | **PI** | **PC** | **PI** | **PC** | **PI** | **PC** | **PI** | **PC** | **PI** |
| **Bio1** | 0.2 | 0.0 | - | - | 0.1 | 1.4 | - | - | 0.2 | 0.1 | - | - | 0.0 | 0.0 | - | - |
| **Bio2** | 0.3 | 0.2 | - | - | 8.3 | 7.4 | - | - | 6.6 | 3.4 | - | - | 0.5 | 0.0 | - | - |
| **Bio3** | 0.2 | 0.1 | 0.2 | 1.4 | 0.5 | 0.3 | 1.9 | 1.6 | 6.2 | 2.3 | 8.3 | 3.9 | 2.2 | 1.8 | 2.0 | 0.5 |
| **Bio4** | 0.2 | 0.2 | 0.2 | 0.1 | 0.0 | 0.0 | 0.0 | 0.0 | 0.0 | 0.0 | 0.0 | 0.0 | 0.2 | 3.2 | 1.0 | 2.2 |
| **Bio5** | 0.2 | 0.0 | - | - | 0.2 | 0.4 | - | - | 5.0 | 2.7 | - | - | 0.5 | 8.6 | - | - |
| **Bio6** | 4.7 | 7.7 | **5.1** | **20.5** | 0.8 | 0.5 | 3.5 | 9.3 | 0.0 | 0.0 | 0.0 | 0.0 | 0.0 | 0.0 | 0.0 | 0.0 |
| **Bio7** | 0.0 | 0.0 | 0.0 | 0.0 | 0.0 | 0.0 | 4.5 | 5.3 | 0.0 | 0.0 | 3.4 | 5.8 | 2.4 | 0.0 | 3.6 | 20.2 |
| **Bio8** | 0.3 | 0.0 | **-** | **-** | 0.4 | 3.2 | **-** | **-** | 0.0 | 0.0 | **-** | **-** | 1.1 | 9.5 | **-** | **-** |
| **Bio9** | 1.9 | 0.0 | 3.0 | 2.4 | **48.5** | **11.7** | **49.9** | **42.5** | 9.4 | 0.0 | **13.1** | **3.0** | **70.3** | **39.0** | **69.9** | **49.9** |
| **Bio10** | 0.1 | 0.0 | - | - | 0.0 | 0.0 | - | - | 0.0 | 0.0 | - | - | 0.0 | 0.0 | - | - |
| **Bio11** | 2.5 | 19.4 | - | - | 0.1 | 3.5 | - | - | 0.1 | 0.0 | - | - | 0.0 | 0.0 | - | - |
| **Bio12** | 0.0 | 0.0 | - | - | 0.0 | 0.0 | - | - | 0.0 | 0.0 | - | - | 0.0 | 0.0 | - | - |
| **Bio13** | 0.0 | 0.0 | - | - | 0.1 | 0.0 | - | - | 0.0 | 0.0 | - | - | 3.3 | 0.0 | - | - |
| **Bio14** | 4.5 | 0.7 | - | - | **13.0** | **6.1** | - | - | 7.9 | 2.5 | - | - | 0.0 | 0.0 | - | - |
| **Bio15** | 3.0 | 0.1 | 3.3 | 3.0 | **23.8** | **46.1** | **24.4** | **32.0** | **15.4** | **0.0** | **15.8** | **0.0** | **16.5** | **31.3** | **16.8** | **18.9** |
| **Bio16** | **2.1** | **42.3** | - | - | 0.4 | 0.0 | - | - | 0.3 | 0.0 | - | - | 0.0 | 0.1 | - | - |
| **Bio17** | 3.0 | 4.1 | - | - | 0.5 | 1.3 | - | - | 0.0 | 0.0 | - | - | 0.0 | 0.0 | - | - |
| **Bio18** | **32.0** | **25.2** | **48.7** | **16.5** | **12.5** | **17.1** | **13.7** | **8.4** | **44.0** | **80.2** | **53.5** | **78.2** | 2.1 | 3.4 | 2.4 | 7.8 |
| **Bio19** | 5.4 | 0.0 | **39.5** | **56.3** | 2.5 | 1.0 | 1.9 | 1.0 | 4.9 | 8.9 | 5.8 | 9.1 | 0.8 | 3.0 | 4.4 | 0.5 |
|  |  |  |  |  |  |  |  |  |  |  |  |  |  |  |  |  |
| **Population groups** | **Dhh01-15, Dhp01,02** | | | | **Dhh01-15** | | | | **Dhh01-08** | | | | **Dhh09-15** | | | |
| **Variable set** | **AV** | | **RV** | | **AV** | | **RV** | | **AV** | | **RV** | | **AV** | | **RV** | |
| **LGM CCSM** | **PC** | **PI** | **PC** | **PI** | **PC** | **PI** | **PC** | **PI** | **PC** | **PI** | **PC** | **PI** | **PC** | **PI** | **PC** | **PI** |
| **Bio1** | 0.2 | 0.0 | - | - | 0.1 | 1.4 | - | - | 0.2 | 0.1 | - | - | 0.0 | 0.0 | - | - |
| **Bio2** | 0.3 | 0.2 | - | - | 8.3 | 7.4 | - | - | 6.6 | 3.4 | - | - | 0.5 | 0.0 | - | - |
| **Bio3** | 0.2 | 0.1 | 0.2 | 1.4 | 0.5 | 0.3 | 1.9 | 1.6 | 6.2 | 2.3 | 8.3 | 3.9 | 2.2 | 1.8 | 2.0 | 0.5 |
| **Bio4** | 0.2 | 0.2 | 0.2 | 0.1 | 0.0 | 0.0 | 0.0 | 0.0 | 0.0 | 0.0 | 0.0 | 0.0 | 0.2 | 3.2 | 1.0 | 2.2 |
| **Bio5** | 0.2 | 0.0 | - | - | 0.2 | 0.4 | - | - | 5.0 | 2.7 | - | - | 0.5 | 8.6 | - | - |
| **Bio6** | 4.2 | 7.7 | 5.1 | 20.5 | 0.8 | 0.5 | 3.5 | 9.3 | 0.0 | 0.0 | 0.0 | 0.0 | 0.0 | 0.0 | 0.0 | 0.0 |
| **Bio7** | 0.0 | 0.0 | 0.0 | 0.0 | 0.0 | 0.0 | 4.5 | 5.3 | 0.0 | 0.0 | 3.4 | 5.8 | 2.4 | 0.0 | **3.6** | **20.2** |
| **Bio8** | 0.3 | 0.0 | **-** | **-** | **0.4** | **3.2** | **-** | **-** | 0.0 | 0.0 | **-** | **-** | 1.1 | 9.5 | **-** | **-** |
| **Bio9** | 1.9 | 0.0 | 3.0 | 2.4 | **48.5** | **11.7** | **49.9** | **42.5** | 9.4 | 0.0 | **13.1** | **3.0** | **70.3** | **39.0** | **69.9** | **49.9** |
| **Bio10** | 0.1 | 0.0 | - | - | 0.0 | 0.0 | - | - | 0.0 | 0.0 | - | - | 0.0 | 0.0 | - | - |
| **Bio11** | 2.5 | 19.4 | - | - | 0.1 | 3.5 | - | - | 0.1 | 0.0 | - | - | 0.0 | 0.0 | - | - |
| **Bio12** | 0.0 | 0.0 | - | - | 0.0 | 0.0 | - | - | 0.0 | 0.0 | - | - | 0.0 | 0.0 | - | - |
| **Bio13** | 0.0 | 0.0 | - | - | 0.1 | 0.0 | - | - | 0.0 | 0.0 | - | - | 3.3 | 0.0 | - | - |
| **Bio14** | 4.5 | 0.7 | - | - | 1.3 | 6.1 | - | - | 7.9 | 2.5 | - | - | 0.0 | 0.0 | - | - |
| **Bio15** | 3.0 | 0.1 | 3.3 | 3.0 | **23.8** | **46.1** | **24.4** | **32.0** | **15.4** | **0.0** | **15.8** | **0.0** | **16.5** | **31.3** | **16.8** | **18.9** |
| **Bio16** | **2.1** | **42.3** | - | - | 0.4 | 0.0 | - | - | 0.3 | 0.0 | - | - | 0.0 | 0.1 | - | - |
| **Bio17** | 3.0 | 4.1 | - | - | 0.5 | 1.3 | - | - | 0.0 | 0.0 | - | - | 0.0 | 0.0 | - | - |
| **Bio18** | **32.0** | **25.2** | **39.5** | **56.3** | **12.5** | **17.1** | **13.7** | **8.4** | **44.0** | **80.2** | **53.5** | **78.2** | 2.1 | 3.4 | 2.4 | 7.8 |
| **Bio19** | **45.4** | **0.0** | **48.7** | **16.5** | 2.5 | 1.0 | 1.9 | 1.0 | 4.9 | 8.9 | 5.8 | 9.1 | 0.8 | 3.0 | 4.4 | 0.5 |
|  |  |  |  |  |  |  |  |  |  |  |  |  |  |  |  |  |
| **Population groups** | **Dhh01-15, Dhp01,02** | | | | **Dhh01-15** | | | | **Dhh01-08** | | | | **Dhh09-15** | | | |
| **Variable set** | **AV** | | **RV** | | **AV** | | **RV** | | **AV** | | **RV** | | **AV** | | **RV** | |
| **LIG** | PC | PI | PC | PI | PC | PI | PC | PI | PC | PI | PC | PI | PC | PI | PC | PI |
| **Bio1** | 0.2 | 0.0 | - | - | 0.1 | 1.7 | - | - | 0.2 | 0.1 | - | - | 0.0 | 0.0 | - | - |
| **Bio2** | 0.3 | 0.2 | - | - | 8.3 | 7.4 | - | - | 6.6 | 3.4 | - | - | 0.5 | 0.0 | - | - |
| **Bio3** | 0.2 | 0.1 | 0.2 | 1.4 | 0.5 | 0.3 | 1.9 | 1.6 | 6.2 | 2.3 | 8.3 | 3.9 | 2.2 | 1.8 | 2.0 | 0.5 |
| **Bio4** | 0.2 | 0.2 | 0.2 | 0.1 | 0.0 | 0.0 | 0.0 | 0.0 | 0.0 | 0.0 | 0.0 | 0.0 | 0.2 | 3.2 | 1.0 | 2.2 |
| **Bio5** | 0.2 | 0.0 | - | - | 0.2 | 0.4 | - | - | 5.0 | 2.7 | - | - | 0.5 | 8.6 | - | - |
| **Bio6** | 4.2 | 7.7 | **5.1** | **20.5** | 0.8 | 0.5 | 3.5 | 9.3 | 0.0 | 0.0 | 0.0 | 0.0 | 0.0 | 0.0 | 0.0 | 0.0 |
| **Bio7** | 0.0 | 0.0 | 0.0 | 0.0 | 0.0 | 0.0 | 4.5 | 5.3 | 0.0 | 0.0 | 3.4 | 5.8 | 2.4 | 0.0 | **3.6** | **20.2** |
| **Bio8** | 0.3 | 0.0 | **-** | **-** | 0.4 | 3.2 | **-** | **-** | 0.0 | 0.0 | **-** | **-** | 1.1 | 9.5 | **-** | **-** |
| **Bio9** | 1.9 | 0.0 | 3.0 | 2.4 | **48.5** | **11.7** | **49.9** | **42.5** | 9.4 | 0.0 | **13.1** | **3.0** | **70.3** | **39.0** | **69.9** | **49.9** |
| **Bio10** | 0.1 | 0.0 | - | - | 0.0 | 0.0 | - | - | 0.0 | 0.0 | - | - | 0.0 | 0.0 | - | - |
| **Bio11** | **2.5** | **19.4** | - | - | 0.1 | 3.5 | - | - | 0.1 | 0.0 | - | - | 0.0 | 0.0 | - | - |
| **Bio12** | 0.0 | 0.0 | - | - | 0.0 | 0.0 | - | - | 0.0 | 0.0 | - | - | 0.0 | 0.0 | - | - |
| **Bio13** | 0.0 | 0.0 | - | - | 0.1 | 0.0 | - | - | 0.0 | 0.0 | - | - | 3.3 | 0.0 | - | - |
| **Bio14** | 4.5 | 0.7 | - | - | 13.0 | 6.4 | - | - | 7.9 | 2.5 | - | - | 0.0 | 0.0 | - | - |
| **Bio15** | 3.0 | 0.1 | 3.3 | 3.0 | **23.8** | **46.1** | **24.4** | **32.0** | **15.4** | **0.0** | **15.8** | **0.0** | **16.5** | **31.3** | **16.8** | **18.9** |
| **Bio16** | **2.1** | **42.3** | - | - | 0.4 | 0.0 | - | - | 0.3 | 0.0 | - | - | 0.0 | 0.1 | - | - |
| **Bio17** | 3.0 | 4.1 | - | - | 0.5 | 1.3 | - | - | 0.0 | 0.0 | - | - | 0.0 | 0.0 | - | - |
| **Bio18** | **32.0** | **25.2** | **39.5** | **56.3** | **12.5** | **17.1** | **13.7** | **8.4** | **44.0** | **80.2** | **53.5** | **78.2** | 2.1 | 3.4 | 2.4 | 7.8 |
| **Bio19** | **45.4** | **0.0** | **48.7** | **16.5** | 2.5 | 1.0 | 1.9 | 1.0 | 4.9 | 8.9 | 5.8 | 9.7 | 0.8 | 3.0 | 4.4 | 0.5 |

| **Table S3.** Area Under the Curve (AUC) for training and test data values, logistic threshold applied and fractional predicted area obtained in the different subsets applied for niche modelling in Maxent. | | | | | |
| --- | --- | --- | --- | --- | --- |
| **Maxent analysis** | **Population groups** | **AUC training** | **AUC test** | **Logistic threshold** | **Fractional predicted area** |
| **Present**  **All 20 variables** | Dhh01-15, Dhp01-02 | 0.963 ± 0.039 | 0.963 ± 0.040 | 0.374 ± 0.042 | 0.044 ± 0.005 |
|  | Dhh01-15 | 0.958 ± 0.074 | 0.958 ± 0.076 | 0.386 ± 0.034 | 0.046 ± 0.005 |
|  | Dhh01-08 | 0.987 ± 0.010 | 0.987 ± 0.011 | 0.484 ± 0.053 | 0.015 ± 0.003 |
|  | Dhh09-15 | 0.937 ± 0.105 | 0.937 ± 0.114 | 0.300 ± 0.094 | 0.093 ± 0.032 |
| **Present**  **Reduced 9 variable set** | Dhh01-15, Dhp01-02 | 0.969 ± 0.035 | 0.969 ± 0.036 | 0.381 ± 0.041 | 0.047 ± 0.007 |
|  | Dhh01-15 | 0.959 ± 0.057 | 0.959 ± 0.059 | 0.458 ± 0.031 | 0.045 ± 0.006 |
|  | Dhh01-08 | 0.989 ± 0.008 | 0.989 ± 0.008 | 0.464 ± 0.048 | 0.038 ± 0.057 |
|  | Dhh09-15 | 0.942 ± 0.090 | 0.942 ± 0.097 | 0.340 ± 0.082 | 0.084 ± 0.028 |
| **LGM CCSM**  **All 20 variables** | Dhh01-15, Dhp01-02 | 0.962 ± 0.039 | 0.962 ± 0.040 | 0.358 ± 0.034 | 0.053 ± 0.005 |
|  | Dhh01-15 | 0.952 ± 0.069 | 0.952 ± 0.071 | 0.460 ± 0.033 | 0.046 ± 0.006 |
|  | Dhh01-08 | 0.987 ± 0.010 | 0.987 ± 0.011 | 0.415 ± 0.040 | 0.020 ± 0.003 |
|  | Dhh09-15 | 0.938 ± 0.105 | 0.938 ± 0.113 | 0.300 ± 0.094 | 0.092 ±0.032 |
| **LGM CCSM**  **Reduced 9 variable set** | Dhh01-15, Dhp01-02 | 0.965 ± 0.039 | 0.965 ± 0.040 | 0.381 ± 0.047 | 0.055 ± 0.006 |
|  | Dhh01-15 | 0.954 ±0.056 | 0.954 ±0.058 | 0.478 ± 0.032 | 0.047 ± 0.006 |
|  | Dhh01-08 | 0.986 ± 0.012 | 0.986 ± 0.013 | 0.391 ±0.044 | 0.026 ± 0.006 |
|  | Dhh09-15 | 0.942 ± 0.090 | 0.942 ± 0.014 | 0.340 ± 0.035 | 0.084 ± 0.006 |
| **LGM MIROC**  **All 20 variables** | Dhh01-15, Dhp01-02 | 0.962 ±0.039 | 0.962 ±0.040 | 0.358 ± 0.034 | 0.053 ± 0.005 |
|  | Dhh01-15 | 0.952 ± 0.069 | 0.952 ± 0.071 | 0.460 ± 0.033 | 0.046 ± 0.006 |
|  | Dhh01-08 | 0.987 ± 0.010 | 0.987 ± 0.011 | 0.415 ± 0.040 | 0.020 ± 0.003 |
|  | Dhh09-15 | 0.938 ± 0.105 | 0.935 ± 0.113 | 0.300 ± 0.094 | 0.092 ± 0.032 |
| **LGM MIROC**  **Reduced 9 variable set** | Dhh01-15, Dhp01-02 | 0.965 ± 0.039 | 0.965 ± 0.040 | 0.381 ± 0.047 | 0.055 ± 0.006 |
|  | Dhh01-15 | 0.954 ± 0.056 | 0.0954 ± 0.058 | 0.478 ± 0.032 | 0.047 ± 0.006 |
|  | Dhh01-08 | 0.986 ± 0.012 | 0.986 ± 0.013 | 0.391 ± 0.044 | 0.026 ± 0.006 |
|  | Dhh09-15 | 0.942 ± 0.090 | 0.942 ± 0.097 | 0.340 ± 0.082 | 0.084 ± 0.028 |
| **LIG**  **All 20 variables** | Dhh01-15, Dhp01-02 | 0.962 ±0.039 | 0.962 ± 0.040 | 0.358 ± 0.034 | 0.056 ± 0.011 |
|  | Dhh01-15 | 0.952 ±0.069 | 0.952 ± 0.071 | 0.452 ± 0.048 | 0.046 ± 0.006 |
|  | Dhh01-08 | 0.987 ± 0.010 | 0.987 ± 0.011 | 0.415 ± 0.040 | 0.039 ± 0.053 |
|  | Dhh09-15 | 0.938 ± 0.105 | 0.933 ± 0.111 | 0.300 ± 0.094 | 0.092 ± 0.032 |
| **LIG**  **Reduced 9 variable set** | Dhh01-15, Dhp01-02 | 0.965 ± 0.039 | 0.965 ± 0.040 | 0.381 ± 0.047 | 0.055 ± 0.006 |
|  | Dhh01-15 | 0.954 ± 0.056 | 0.954 ± 0.058 | 0.478 ±0.032 | 0.047 ± 0.006 |
|  | Dhh01-08 | 0.986 ± 0.012 | 0.986 ± 0.013 | 0.391 ± 0.045 | 0.026 ± 0.006 |
|  | Dhh09-15 | 0.942 ± 0.090 | 0.942 ± 0.097 | 0.340 ± 0.082 | 0.084 ± 0.028 |

3

2

1

0

-1

**PC2 (26.14%)**

3

2

1

0

-1

-2

Dhp02

Dhp01

Dhh15

Dhh14

Dhh13

Dhh12

Dhh11

Dhh10

Dhh09

Dhh08

Dhh07

Dhh06

Dhh05

Dhh04

Dhh03

Dhh02

Dhh01

**PC1 (47.81%)**

| Variable | PC1 | PC2 | PC3 |
| --- | --- | --- | --- |
| Alt | **0.966** | -0.010 | 0.174 |
| Bio1 | -0.236 | **0.690** | **-0.609** |
| Bio2 | **-0.763** | -0.181 | **0.609** |
| Bio3 | -0.499 | **0.671** | -0.380 |
| Bio4 | -0.379 | **-0.542** | -0.663 |
| Bio5 | -0.051 | **0.912** | 0.395 |
| Bio6 | -0.438 | **0.672** | **0.587** |
| Bio7 | -0.295 | **-0.785** | **0.536** |
| Bio8 | -0.177 | **0.966** | 0.158 |
| Bio9 | **-0.522** | **-0.665** | 0.487 |
| Bio10 | **-0.520** | 0.314 | **0.781** |
| Bio11 | **-0.577** | 0.360 | **0.731** |
| Bio12 | **-0.622** | **-0.673** | 0.371 |
| Bio13 | **0.968** | 0.003 | 0.230 |
| Bio14 | **0.941** | 0.058 | 0.230 |
| Bio15 | **0.942** | -0.012 | 0.255 |
| Bio16 | **-0.846** | -0.093 | -0.159 |
| Bio17 | **0.954** | 0.026 | 0.238 |
| Bio18 | **0.907** | -0.022 | 0.249 |
| Bio19 | **0.935** | -0.085 | 0.236 |

**Figure S1.** Two-Dimensional plot of Principal Coordinate Analysis (PCA) constructed with the bioclimatic and altitudinal variables obtained from WorldClim for the populations of *Dioscorea humilis*. The proportion of explained variance by each PC axis is indicated in brackets. The coefficient of contribution of each variable to the positive and negative extremes of the three first PC axes is shown. Those variables that most contribute to each case are indicated in bold.
