# Supplementary material for: Latitudinal Environmental Niches and Riverine Barriers Shaped the Phylogeography of the Central Chilean Endemic Dioscorea humilis (Dioscoreaceae)
Source: PLoS One. 2014 Oct 8;9(10):e110029. doi: 10.1371/journal.pone.0110029 (PMC4190404; doi:10.1371/journal.pone.0110029)
Supplement: Appendix S2 — Microsatellite allele frequencies, Bayesian estimation of genetic clusters and plastid haplotype frequencies in populations of Dioscorea humilis . (DOC) [file pone.0110029.s002.doc]

| **Table S4.** Allele frequencies for eight microsatellite loci in 17 populations of *Dioscorea humilis.* Allele sizes are indicated in bp. Numbers in brackets indicate sample sizes. | | | | | | | | | | | | | | | | | |
| --- | --- | --- | --- | --- | --- | --- | --- | --- | --- | --- | --- | --- | --- | --- | --- | --- | --- |
| **Taxon** | ***Dioscorea humilis* subsp. *humilis*** | | | | | | | | | | | | | | | ***Dioscorea humilis* subsp. *polyanthes*** | |
| **Locus/population**  **(*N*)** | **Dhh01**  (30) | **Dhh02**  (28) | **Dhh03**  (30) | **Dhh04**  (30) | **Dhh05**  (36) | **Dhh06**  (31) | **Dhh07**  (36) | **Dhh08**  (36) | **Dhh09**  (36) | **Dhh10**  (36) | **Dhh11**  (30) | **Dhh12**  (29) | **Dhh13**  (36) | **Dhh14**  (36) | **Dhh15**  (28) | **Dhp01**  (35) | **Dhp02**  (35) |
| **B812** |  |  |  |  |  |  |  |  |  |  |  |  |  |  |  |  |  |
| 186 | 0 | 0 | 0 | 0 | 0 | 0 | 0 | 0 | 0 | 0 | 0 | 0 | 0 | 0 | 0 | 0.0143 | 0 |
| 189 | 0 | 0 | 0 | 0 | 0 | 0 | 0 | 0 | 0 | 0 | 0 | 0 | 0 | 0 | 0 | 0.0143 | 0 |
| 192 | 0 | 0 | 0 | 0 | 0 | 0 | 0 | 0 | 0 | 0 | 0 | 0 | 0.0139 | 0 | 0 | 0 | 0 |
| 195 | 0 | 0 | 0 | 0 | 0 | 0 | 0 | 0 | 0 | 0 | 0 | 0 | 0 | 0 | 0 | 0.1000 | 0 |
| 198 | 0.1667 | 0.4286 | 0.3667 | 0.3333 | 0.3429 | 0.2500 | 0.4722 | 0.3857 | 0.0143 | 0 | 0.0167 | 0 | 0 | 0 | 0 | 0.5571 | 0.6143 |
| 201 | 0 | 0 | 0 | 0 | 0 | 0 | 0 | 0 | 0 | 0 | 0 | 0 | 0 | 0 | 0 | 0.2000 | 0.1857 |
| 204 | 0 | 0 | 0 | 0 | 0 | 0 | 0 | 0 | 0 | 0 | 0 | 0 | 0 | 0 | 0 | 0.0571 | 0.1429 |
| 207 | 0 | 0 | 0 | 0 | 0 | 0 | 0 | 0 | 0.0571 | 0.0694 | 0.0167 | 0.0179 | 0.0139 | 0.0143 | 0 | 0.0571 | 0.0571 |
| 210 | 0.8000 | 0.5714 | 0.6333 | 0.6667 | 0.6571 | 0.7500 | 0.5278 | 0.6143 | 0.9286 | 0.9306 | 0.8667 | 0.9821 | 0.9583 | 0.9714 | 1.000 | 0 | 0 |
| 216 | 0 | 0 | 0 | 0 | 0 | 0 | 0 | 0 | 0 | 0 | 0 | 0 | 0 | 0.0143 | 0 | 0 | 0 |
| 219 | 0.0333 | 0 | 0 | 0 | 0 | 0 | 0 | 0 | 0 | 0 | 0 | 0 | 0 | 0 | 0 | 0 | 0 |
| 222 | 0 | 0 | 0 | 0 | 0 | 0 | 0 | 0 | 0 | 0 | 0.100 | 0 | 0.0139 | 0 | 0 | 0 | 0 |
| **B809** |  |  |  |  |  |  |  |  |  |  |  |  |  |  |  |  |  |
| 124 | 0 | 0 | 0 | 0 | 0 | 0 | 0.0139 | 0 | 0 | 0 | 0 | 0 | 0 | 0 | 0 | 0 | 0 |
| 142 | 0 | 0.0179 | 0 | 0.0333 | 0.2222 | 0 | 0.2083 | 0.3333 | 0.0278 | 0.0139 | 0 | 0 | 0 | 0 | 0 | 0 | 0 |
| 145 | 0 | 0 | 0 | 0 | 0 | 0 | 0.0139 | 0.0139 | 0 | 0 | 0 | 0.0517 | 0 | 0.0278 | 0.3393 | 0 | 0 |
| 148 | 0.1000 | 0 | 0.1167 | 0 | 0.0139 | 0.0323 | 0.0694 | 0.0139 | 0.5833 | 0.8333 | 0.2333 | 0.1379 | 0.1111 | 0.0278 | 0.1071 | 0 | 0 |
| 151 | 0.8667 | 0.9821 | 0.8667 | 0.9667 | 0.7361 | 0.9677 | 0.6944 | 0.6389 | 0.3889 | 0.1528 | 0.7667 | 0.8103 | 0.8889 | 0.9444 | 0.5536 | 0.9857 | 1.0000 |
| 154 | 0.0333 | 0 | 0.0167 | 0 | 0.0278 | 0 | 0 | 0 | 0 | 0 | 0 | 0 | 0 | 0 | 0 | 0.0143 | 0 |
| **H442** |  |  |  |  |  |  |  |  |  |  |  |  |  |  |  |  |  |
| 141 | 0 | 0 | 0 | 0 | 0 | 0 | 0 | 0 | 0 | 0 | 0.0167 | 0 | 0 | 0.0469 | 0 | 0 | 0.0143 |
| 143 | 0 | 0 | 0 | 0 | 0 | 0 | 0 | 0 | 0 | 0 | 0 | 0 | 0 | 0 | 0 | 0.0143 | 0.0429 |
| 145 | 0 | 0 | 0 | 0 | 0 | 0 | 0 | 0 | 0 | 0 | 0 | 0 | 0 | 0 | 0 | 0 | 0.0143 |
| 149 | 0 | 0 | 0 | 0 | 0 | 0 | 0 | 0.0139 | 0 | 0 | 0 | 0 | 0 | 0 | 0 | 0 | 0 |
| 151 | 0 | 0 | 0 | 0 | 0 | 0 | 0 | 0.0139 | 0 | 0 | 0 | 0 | 0 | 0.0313 | 0 | 0 | 0 |
| 153 | 0 | 0 | 0 | 0 | 0 | 0 | 0 | 0.0139 | 0 | 0 | 0 | 0 | 0 | 0 | 0 | 0 | 0 |
| 155 | 0.0500 | 0.0179 | 0 | 0 | 0.0147 | 0 | 0 | 0.0139 | 0 | 0 | 0.0167 | 0 | 0 | 0.0313 | 0 | 0 | 0 |
| 157 | 0.2000 | 0 | 0.0667 | 0.0167 | 0.1029 | 0 | 0.1667 | 0.1667 | 0.1719 | 0.0429 | 0.2667 | 0.0345 | 0.2222 | 0.0625 | 0.0714 | 0.2571 | 0.2857 |
| 159 | 0.0167 | 0.0714 | 0.0500 | 0.0667 | 0.1765 | 0.1071 | 0.2083 | 0.2639 | 0.2656 | 0.3857 | 0.2667 | 0.3621 | 0.2500 | 0.4844 | 0.4286 | 0.2143 | 0.1000 |
| 161 | 0.0667 | 0.0179 | 0.0500 | 0.0833 | 0.0147 | 0 | 0.0139 | 0 | 0.0625 | 0.1143 | 0 | 0.1034 | 0.0417 | 0 | 0.0179 | 0.0143 | 0 |
| 163 | 0.0167 | 0.2321 | 0.1333 | 0.1667 | 0.0441 | 0.2321 | 0.1667 | 0.0556 | 0.1094 | 0.0571 | 0.0667 | 0.0345 | 0.1944 | 0.1094 | 0.2143 | 0 | 0.0286 |
| 165 | 0.1167 | 0.0536 | 0.0833 | 0.0167 | 0.0147 | 0 | 0 | 0 | 0.0156 | 0.0143 | 0.0333 | 0 | 0 | 0.0313 | 0.0357 | 0.1143 | 0.0857 |
| 167 | 0.0333 | 0.0179 | 0.0833 | 0 | 0.0147 | 0.0536 | 0 | 0 | 0 | 0.0286 | 0.0167 | 0 | 0 | 0.0156 | 0 | 0.0143 | 0.0429 |
| 169 | 0 | 0.0179 | 0.0333 | 0 | 0.0735 | 0.0357 | 0 | 0.0278 | 0.0156 | 0.0714 | 0.0167 | 0.0345 | 0 | 0 | 0 | 0.1000 | 0.1571 |
| 171 | 0.3000 | 0.1964 | 0.2333 | 0.1833 | 0.3235 | 0.1786 | 0.0417 | 0.1111 | 0.0469 | 0.0571 | 0 | 0.1724 | 0.0139 | 0.0313 | 0.0357 | 0.0286 | 0.0571 |
| 173 | 0.1000 | 0.1071 | 0.0333 | 0.0833 | 0.0882 | 0.0714 | 0.3056 | 0.1528 | 0.1563 | 0.200 | 0.2167 | 0.1379 | 0.1667 | 0.1250 | 0.0893 | 0.0143 | 0.0143 |
| 175 | 0.0333 | 0.0179 | 0.0500 | 0.0500 | 0.0441 | 0.0179 | 0.0278 | 0.0972 | 0.0313 | 0 | 0.0500 | 0.0172 | 0.0139 | 0 | 0.0893 | 0.2286 | 0.1429 |
| 177 | 0.0167 | 0.0179 | 0.0333 | 0.1500 | 0 | 0.0893 | 0.0417 | 0.0278 | 0.0469 | 0 | 0 | 0.0172 | 0.0139 | 0 | 0 | 0 | 0 |
| 179 | 0.0167 | 0.2143 | 0.0667 | 0.1833 | 0.0882 | 0.1429 | 0 | 0.0139 | 0 | 0.0286 | 0 | 0.0862 | 0.0139 | 0.0156 | 0 | 0 | 0 |
| 181 | 0 | 0.0179 | 0.0167 | 0 | 0 | 0.0714 | 0.0278 | 0.0278 | 0.0781 | 0 | 0.0167 | 0 | 0.0139 | 0 | 0 | 0 | 0 |
| 183 | 0 | 0 | 0.0667 | 0 | 0 | 0 | 0 | 0 | 0 | 0 | 0.0167 | 0 | 0 | 0 | 0.0179 | 0 | 0 |
| 185 | 0.0333 | 0 | 0 | 0 | 0 | 0 | 0 | 0 | 0 | 0 | 0 | 0 | 0.0278 | 0.0156 | 0 | 0 | 0 |
| 187 | 0 | 0 | 0 | 0 | 0 | 0 | 0 | 0 | 0 | 0 | 0 | 0 | 0.0278 | 0 | 0 | 0 | 0 |
| 189 | 0 | 0 | 0 | 0 | 0 | 0 | 0 | 0 | 0 | 0 | 0 | 0 | 0 | 0 | 0 | 0 | 0.0143 |
| **B628** |  |  |  |  |  |  |  |  |  |  |  |  |  |  |  |  |  |
| 142 | 0 | 0 | 0 | 0 | 0 | 0 | 0 | 0 | 0 | 0 | 0 | 0 | 0 | 0 | 0.0179 | 0 | 0 |
| 145 | 0 | 0.1071 | 0.0167 | 0.0167 | 0.0278 | 0.0323 | 0.0278 | 0.0139 | 0.0139 | 0.0143 | 0 | 0 | 0 | 0 | 0 | 0 | 0 |
| 148 | 0.3667 | 0.0893 | 0.2667 | 0.1833 | 0.2500 | 0.2581 | 0.2361 | 0.3611 | 0.4583 | 0.3286 | 0.4500 | 0.4483 | 0.4722 | 0.375 | 0.4464 | 0.4000 | 0.2429 |
| 151 | 0.4833 | 0.7857 | 0.700 | 0.6667 | 0.6806 | 0.5645 | 0.7361 | 0.5139 | 0.3889 | 0.6429 | 0.4667 | 0.5172 | 0.4722 | 0.5278 | 0.5000 | 0.6000 | 0.6429 |
| 154 | 0.1333 | 0 | 0.0167 | 0.1333 | 0 | 0.1452 | 0 | 0.0694 | 0.1111 | 0.0143 | 0.0833 | 0.0345 | 0.0278 | 0.0972 | 0.0357 | 0 | 0.0857 |
| 157 | 0.0167 | 0.0179 | 0 | 0 | 0 | 0 | 0 | 0 | 0.0278 | 0 | 0 | 0 | 0.0278 | 0 | 0 | 0 | 0.0286 |
| 160 | 0 | 0 | 0 | 0 | 0.0417 | 0 | 0 | 0.0417 | 0 | 0 | 0 | 0 | 0 | 0 | 0 | 0 | 0 |
| **H422** |  |  |  |  |  |  |  |  |  |  |  |  |  |  |  |  |  |
| 107 | 0 | 0 | 0 | 0 | 0 | 0 | 0 | 0 | 0 | 0.1111 | 0 | 0 | 0 | 0 | 0 | 0 | 0 |
| 110 | 0 | 0 | 0 | 0 | 0 | 0 | 0 | 0 | 0 | 0 | 0.0167 | 0 | 0 | 0.0417 | 0 | 0 | 0 |
| 113 | 0 | 0 | 0 | 0 | 0 | 0 | 0 | 0.0147 | 0 | 0 | 0 | 0 | 0.0429 | 0.0556 | 0.0179 | 0 | 0 |
| 116 | 0.1000 | 0.1964 | 0.3000 | 0.3667 | 0.1806 | 0.3387 | 0.0556 | 0.1618 | 0.0441 | 0.1806 | 0.0167 | 0.0172 | 0.0571 | 0.1111 | 0 | 0.0286 | 0 |
| 119 | 0.5000 | 0.5536 | 0.4000 | 0.4333 | 0.3750 | 0.5161 | 0.4861 | 0.2794 | 0.4118 | 0.4167 | 0.5333 | 0.2241 | 0.4571 | 0.4444 | 0.2143 | 0.8857 | 0.8714 |
| 122 | 0.3667 | 0.2500 | 0.2667 | 0.1833 | 0.3611 | 0.1290 | 0.2361 | 0.4853 | 0.2647 | 0.1528 | 0.2500 | 0.4828 | 0.2571 | 0.2500 | 0.5357 | 0.0857 | 0.1143 |
| 125 | 0.0333 | 0 | 0.0333 | 0.0167 | 0.0833 | 0.0161 | 0.2222 | 0.0588 | 0.2500 | 0.1389 | 0.1333 | 0.2759 | 0.1857 | 0.0833 | 0.2321 | 0 | 0 |
| 128 | 0 | 0 | 0 | 0 | 0 | 0 | 0 | 0 | 0.0294 | 0 | 0.0333 | 0 | 0 | 0 | 0 | 0 | 0.0143 |
| 131 | 0 | 0 | 0 | 0 | 0 | 0 | 0 | 0 | 0 | 0 | 0.0167 | 0 | 0 | 0.0139 | 0 | 0 | 0 |
| **B322** |  |  |  |  |  |  |  |  |  |  |  |  |  |  |  |  |  |
| 181 | 0 | 0 | 0 | 0 | 0 | 0 | 0 | 0 | 0.0606 | 0 | 0 | 0 | 0 | 0 | 0 | 0 | 0 |
| 184 | 0 | 0.0357 | 0.0167 | 0 | 0 | 0.0645 | 0 | 0 | 0 | 0.1212 | 0.0517 | 0 | 0.0417 | 0.0441 | 0.0536 | 0 | 0.0143 |
| 187 | 1.0000 | 0.9464 | 0.9833 | 0.9500 | 1 | 0.6613 | 0.9861 | 0.9861 | 0.2879 | 0.4697 | 0.4655 | 0 | 0.2778 | 0.1618 | 0.1964 | 0.0441 | 0 |
| 190 | 0 | 0.0179 | 0 | 0.0500 | 0 | 0.2742 | 0.0139 | 0.0139 | 0.2879 | 0.1970 | 0.2414 | 1.0000 | 0.4583 | 0.1912 | 0.25 | 0.3529 | 0.3571 |
| 193 | 0 | 0 | 0 | 0 | 0 | 0 | 0 | 0 | 0.0152 | 0.1970 | 0.0862 | 0 | 0.1528 | 0.2206 | 0.3929 | 0.5441 | 0.5286 |
| 196 | 0 | 0 | 0 | 0 | 0 | 0 | 0 | 0 | 0 | 0.0152 | 0.0517 | 0 | 0.0278 | 0.1324 | 0 | 0.0588 | 0.0857 |
| 198 | 0 | 0 | 0 | 0 | 0 | 0 | 0 | 0 | 0 | 0 | 0.0172 | 0 | 0 | 0 | 0 | 0 | 0 |
| 199 | 0 | 0 | 0 | 0 | 0 | 0 | 0 | 0 | 0.0152 | 0 | 0.0517 | 0 | 0.0278 | 0.2353 | 0 | 0 | 0 |
| 202 | 0 | 0 | 0 | 0 | 0 | 0 | 0 | 0 | 0.0455 | 0 | 0.0172 | 0 | 0 | 0.0147 | 0.1071 | 0 | 0.0143 |
| 205 | 0 | 0 | 0 | 0 | 0 | 0 | 0 | 0 | 0 | 0 | 0.0172 | 0 | 0.0139 | 0 | 0 | 0 | 0 |
| 211 | 0 | 0 | 0 | 0 | 0 | 0 | 0 | 0 | 0.2879 | 0 | 0 | 0 | 0 | 0 | 0 | 0 | 0 |
| **B744** |  |  |  |  |  |  |  |  |  |  |  |  |  |  |  |  |  |
| 203 | 0.1667 | 0 | 0 | 0 | 0 | 0 | 0 | 0 | 0 | 0 | 0 | 0 | 0 | 0 | 0 | 0 | 0 |
| 215 | 0.0167 | 0 | 0 | 0 | 0 | 0 | 0 | 0 | 0 | 0 | 0 | 0 | 0 | 0 | 0 | 0 | 0 |
| 224 | 0 | 0 | 0 | 0 | 0 | 0.0161 | 0 | 0 | 0 | 0 | 0 | 0 | 0 | 0 | 0 | 0.1571 | 0.1571 |
| 227 | 0 | 0.5000 | 0.0167 | 0.3000 | 0.0972 | 0.3871 | 0.0286 | 0.0139 | 0 | 0.0972 | 0 | 0 | 0 | 0 | 0.0536 | 0.1000 | 0.1000 |
| 230 | 0.2167 | 0.0714 | 0.0833 | 0.0333 | 0.0417 | 0.1290 | 0.3286 | 0.3750 | 0.0694 | 0.2222 | 0.1833 | 0.3966 | 0.4028 | 0.1364 | 0.3214 | 0.5571 | 0.5429 |
| 233 | 0.6000 | 0.3571 | 0.9000 | 0.6667 | 0.8611 | 0.4516 | 0.6429 | 0.6111 | 0.9306 | 0.6667 | 0.8167 | 0.6034 | 0.5972 | 0.8636 | 0.625 | 0.1857 | 0.2000 |
| 245 | 0 | 0.0714 | 0 | 0 | 0 | 0.0161 | 0 | 0 | 0 | 0.0139 | 0 | 0 | 0 | 0 | 0 | 0 | 0 |
| **B633** |  |  |  |  |  |  |  |  |  |  |  |  |  |  |  |  |  |
| 128 | 0 | 0 | 0 | 0 | 0 | 0 | 0 | 0 | 0 | na | 0 | 0 | 0 | 0 | 0 | 0.4706 | 0.2429 |
| 131 | 1.0000 | 1.0000 | 1.0000 | 1.0000 | 1.0000 | 1.0000 | 1.0000 | 1.0000 | 1.0000 | na | 1.0000 | 1.0000 | 1.0000 | 0.9833 | 0.8654 | 0.5294 | 0.7571 |
| 137 | 0 | 0 | 0 | 0 | 0 | 0 | 0 | 0 | 0 | na | 0 | 0 | 0 | 0.0167 | 0.1346 | 0 | 0 |

***K***

Δ***K***


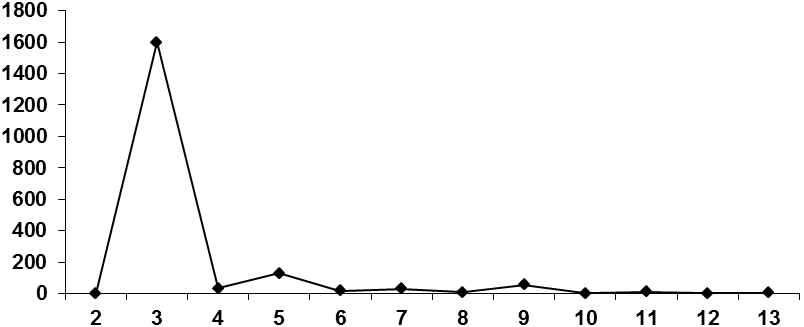


**Figure S2**. Estimation of the most likely number of genetic clusters according to Evanno *et al.* (2005) showing maximum peaks of ∆*K* values at *K* = 3 and *K* = 5, indicating that those are the optimal solutions for *K* given the data.

**Reference**

Evanno, G., Regnaut, S. & Goudet, J. (2005) Detecting the number of clusters of individuals using the software STRUCTURE: a simulation study. *Molecular Ecology*, **14**, 2611-2620.

| **Table S5.** Distribution of plastid haplotypes obtained from independent cpDNA regions *trn*T-*trn*L and *trn*L*-trn*F, and from the linear combination of the two cpDNA regions in the populations of *Dioscorea humilis*. Haplotypes are designated in roman numbers. The numbers in the cells indicate the number of individuals presenting a given haplotype. GB, GenBank accession numbers. | | | | | | | | | | | | | | | | | | | | |
| --- | --- | --- | --- | --- | --- | --- | --- | --- | --- | --- | --- | --- | --- | --- | --- | --- | --- | --- | --- | --- |
| **DNA region** | *trn*T*-trn*L | | | | | | | | | | | | | | | | | | | |
| **Taxon** | ***Dioscorea humilis* subsp. *humilis*** | | | | | | | | | | | | | | |  | ***D. humilis* subsp. *polyanthes*** | |  |  |
| **Hapl./Pop.** | **Dhh01** | **Dhh02** | **Dhh03** | **Dhh04** | **Dhh05** | **Dhh06** | **Dhh07** | **Dhh08** | **Dhh09** | **Dhh10** | **Dhh11** | **Dhh12** | **Dhh13** | **Dhh14** | **Dhh15** |  | **Dhp01** | **Dhp02** | **Total** | **GB** |
| **I** | 5 | - | - | - | - | - | - | - | - | - | - | - | - | - | - |  | - | - | 5 | KF357945 |
| **II** | - | 2 | 2 | 4 | 4 | 4 | 3 | 3 | 2 | - | 2 | 2 | 3 | 4 | 3 |  | - | 6 | 44 | KF357946 |
| **III** | - | - | - | - | - | - | - | - | - | 2 | - | - | - | - | - |  | - | - | 2 | KF357947 |
| **IV** | - | - | - | - | - | - | - | - | - | - | - | - | - | - | - |  | 3 | - | 3 | KF357948 |
| **Total** | 5 | 2 | 2 | 4 | 4 | 4 | 3 | 3 | 2 | 2 | 2 | 2 | 3 | 4 | 3 |  | 3 | 6 | 54 |  |
| **DNA region** | *trn*L*-trn*F | | | | | | | | | | | | | | | | | | | |
| **Taxon** | ***Dioscorea humilis* subsp. *humilis*** | | | | | | | | | | | | | | |  | ***D. humilis* subsp. *polyanthes*** | |  |  |
| **Hapl./Pop.** | **Dhh01** | **Dhh02** | **Dhh03** | **Dhh04** | **Dhh05** | **Dhh06** | **Dhh07** | **Dhh08** | **Dhh09** | **Dhh10** | **Dhh11** | **Dhh12** | **Dhh13** | **Dhh14** | **Dhh15** |  | **Dhp01** | **Dhp02** | **Total** | **GB** |
| **I** | 4 | - | - | - | - | - | - | - | - | - | - | - | - | - | - |  | - | - | 4 | KF357949 |
| **II** | 1 | - | - | - | - | - | - | - | - | - | - | - | - | - | - |  | - | - | 1 | KF357950 |
| **III** | - | 2 | - | 4 | - | - | - | - | - | - | - | - | - | - | - |  | - | - | 6 | KF357951 |
| **IV** | - | - | 2 | - | 4 | 4 | 3 | 3 | 2 | - | 2 | 2 | 3 | 2 | 2 |  | 3 | 6 | 39 | KF357952 |
| **V** | - | - | - | - | - | - | - | - | - | 2 | - | - | - | - | - |  | - | - | 2 | KF357953 |
| **VI** | - | - | - | - | - | - | - | - | - | - | - | - | - | 2 | - |  | - | - | 2 | KF357954 |
| **VII** | - | - | - | - | - | - | - | - | - | - | - | - | - | - | 1 |  | - | - | 1 | KF357955 |
| **Total** | 5 | 2 | 2 | 4 | 4 | 4 | 3 | 3 | 2 | 2 | 2 | 2 | 3 | 4 | 3 |  | 3 | 6 | 54 |  |
| **DNA region** | Combined: *trn*T*-trn*L*+trn*L-*trn*F | | | | | | | | | | | | | | | | | | | |
| **Taxon** | ***Dioscorea humilis* subsp. *humilis*** | | | | | | | | | | | | | | |  | ***D, humilis* subsp. *polyanthes*** | |  |  |
| **Hapl./Pop.** | **Dhh01** | **Dhh02** | **Dhh03** | **Dhh04** | **Dhh05** | **Dhh06** | **Dhh07** | **Dhh08** | **Dhh09** | **Dhh10** | **Dhh11** | **Dhh12** | **Dhh13** | **Dhh14** | **Dhh15** |  | **Dhp01** | **Dhp02** | **Total** | **GB** |
| **I** | 4 | - | - | - | - | - | - | - | - | - | - | - | - | - | - |  | - | - | 4 | KF357945+ KF357949 |
| **II** | 1 | - | - | - | - | - | - | - | - | - | - | - | - | - | - |  | - | - | 1 | KF357945+ KF357950 |
| **III** | - | 2 | - | 4 | - | - | - | - | - | - | - | - | - | - | - |  | - | - | 6 | KF357946+ KF357951 |
| **IV** | - | - | 2 | - | 4 | 4 | 3 | 3 | 2 | - | 2 | 2 | 3 | 2 | 2 |  | - | 6 | 35 | KF357946+ KF357952 |
| **V** | - | - | - | - | - | - | - | - | - | 2 | - | - | - | - | - |  | - | - | 2 | KF357947+ KF357953 |
| **VI** | - | - | - | - | - | - | - | - | - | - | - | - | - | 2 | - |  | - | - | 2 | KF357946+ KF357954 |
| **VII** | - | - | - | - | - | - | - | - | - | - | - | - | - | - | 1 |  | - | - | 1 | KF357946+ KF357955 |
| **VIII** | - | - | - | - | - | - | - | - | - | - | - | - | - | - | - |  | 3 | - | 3 | KF357948+ KF357952 |
| **Total** | 5 | 2 | 2 | 4 | 4 | 4 | 3 | 3 | 2 | 2 | 2 | 2 | 3 | 4 | 3 |  | 3 | 6 | 54 |  |
