# Supplementary material for: Latitudinal Environmental Niches and Riverine Barriers Shaped the Phylogeography of the Central Chilean Endemic Dioscorea humilis (Dioscoreaceae)
Source: PLoS One. 2014 Oct 8;9(10):e110029. doi: 10.1371/journal.pone.0110029 (PMC4190404; doi:10.1371/journal.pone.0110029)
Supplement: Appendix S3 — Comparison of microsatellite genetic diversity in yam species. (DOC) [file pone.0110029.s003.doc]

| **Table S6.** Comparison of microsatellite genetic diversity in *Dioscorea*. | | | | | | | | |
| --- | --- | --- | --- | --- | --- | --- | --- | --- |
| **Species** | ***Range*** | **Habit** | ***Seeds*** | ***Vegetative reproduction*** | ***A**** | ***H*_O_*** | ***H*_E_*** | **Source** |
| *D. abyssinica* Hochst. & Kunth | Tropical Africa | Climber | Winged | Tuber fragments | 19.4 | 0.64 | - | Scarcelli *et al.*, 2005 |
| *D. alata* L. | Pantropical cultured | Climber | Winged | Aerial tubers | 7.46 | 0.469 | - | Obidiegwu *et al.* 2009a |
| *D. biloba* (Phil.) Caddick & Wilkin | Chilean narrow endemic | Dwarf, creeping | Wingless | Absent | 5.14-7.29 | 0.345-0.686 | 0.458-0.706 | Viruel *et al.*, 2012. |
| *D. bulbifera* L. | Pantropical cultured | Climber | Winged | Aerial tubers | 7.30-8.86 | 0.722-0.733 | - | Yan *et al*., 2014 |
| *D. cayenensis* Lam.*/D. rotundata* Poir. | Pantropical cultured | Climber | Winged | Tuber fragments | 8.06 | 0.563 | - | Obidiegwu *et al.* 2009b |
| *D. chouardii* Gaussen | NE Spain narrow endemic | Dwarf, creeping | Wingless | Absent | 1.70 | 0.14 | 0.13-0.14 | Segarra-Moragues *et al.*, 2005 |
| *D. humilis* Colla | Chilean endemic | Dwarf, creeping | Wingless | Absent | 3.25-5.00 | 0.350-0.598 | 0.357-0.521 | This study |
| *D. japonica* Thunb. | SE Asia | climber | Winged | Aerial tubers | 3-9 | - | 0.461-0.851 | Mizuki *et al.*, 2005 |
| *D. praehensilis* | Tropical Africa | Climber | Winged | Tuber fragments | 14.6 | 0.660 | - | Scarcelli *et al.* 2005 |
| *D. pyrenaica* Bubani & Bordère *ex* Gren. | NE Spain narrow endemic | Dwarf, creeping | Wingless | Absent | 1.56-3.22 | 0.122-0.232 | 0.129-0.257 | Segarra-Moragues *et al.* 2007 |
| *D. tokoro* Makino | SE Asia | Climber | Winged | Rhizome fragments | 6.2 | 0.540 | 0.680 | Terauchi & Konuma, 1994 |
| *D. trifida* L.f. | Pantropical cultured | Climber | Winged | Tuber fragments | 6.0 | - | 0.600 | Hochu *et al.*, 2006 |
| *D. rotundata* Poir | Tropical Africa | Climber | Winged | Tuber fragments | 11.5 | 0.58 | - | Scarcelli *et al.*, 2005 |
| *D. zingiberensis* C.H. Wright | SE Asia | Climber | Winged | Rhizome fragments | 9.53 | 0.328 | 0.750 | Yan *et al*., 2013 |
| **A*, average number of alleles; *H*_O_ and *H*_E_, observed and expected heterozygosities, respectively. | | | | | | | | |

**References**

Hochu, I., Santoni, S. & Bousalem, M. (2006) Isolation, characterization and cross-species amplification of microsatellite DNA loci in the tropical American yam *Dioscorea trifi*da. *Molecular Ecology Notes*, **6**, 137-140.

Mizuki I, Tani N, Ishida K, Tsumura Y. 2005. Development and characterization of microsatellite markers in a clonal plant, *Dioscorea japonica* Thunb. *Molecular Ecology Notes*, **5**, 721-723.

Obidiegwu, J., Aiedu, R., Ene-Obong, E.E., Muoneke, C.O. & Kolesnikova-Allen, M. (2009a) Genetic characterization of some water yam (*Dioscorea alata* L.) accessions in West Africa with simple sequence repeats. *International Journal of Food, Agriculture and Environment*, **7**, 634-638.

Obidiegwu, J., Kolesnikova-Allen, M., Ene-Obong, E.E., Mouneke, C.O. & Asiedu, R. (2009b) SSR markers reveal diversity in Guinea yam (*Dioscorea cayenensis*/*D. rotundata*) core set. *African Journal of Biotechnology*, **8**, 2730-2739.

Scarcelli, N., Daïnou, O., Agblanga, C., Tostain, S. & Pham, J.-L. (2005) Segregation patterns of isozyme loci and microsatellite markers show the diploidy of African yam *Dioscorea rotundata* (2*n* = 40). *Theoretical and Applied Genetics*, **111**, 226-232.

Segarra-Moragues, J.G., Palop-Esteban, M., González-Candelas, F., & Catalán, P. (2005) On the verge of extinction: genetics of the Critically Endangered Iberian plant species, *Borderea chouardii* (Dioscoreaceae) and implications for conservation management. *Molecular Ecology*, **14**, 969-982.

Segarra-Moragues, J.G., Palop-Esteban, M., González-Candelas, F. & Catalán, P. (2007) Nunatak survival *vs*. tabula rasa in the Central Pyrenees: a study on the endemic plant species *Borderea pyrenaica* (Dioscoreaceae). *Journal of Biogeography*, **34**, 1893-1906.

Terauchi, R. & Konuma A. (1994) Microsatellite polymorphism in *Dioscorea tokoro*, a wild yam species. *Genome*, **37**, 794-801.

Viruel, J., Catalán, P. & Segarra-Moragues, J.G. (2012) Disrupted phylogeographical SSR and cpDNA patterns indicate a vicariance rather than long-distance dispersal origin for the disjunct distribution of the Chilean endemic *Dioscorea biloba* (Dioscoreaceae) around the Atacama Desert. *Journal of Biogeography*, **39**, 1073-1085.

Yan, Q.-Q., Sun, X.-Q., Guo, J.-L., Hang, Y.-Y. & Li, M.-M. (2013) Development of polymorphic microsatellite markers for *Dioscorea zingiberensis* and cross-amplification in other *Dioscorea* species. *Genetics and Molecular Research*, **12,** 3788-3792.

Yan, Q.-Q., Li, Y., Sun, X.-Q., Guo, J.-L., Hang, Y.-Y. & Li, M.-M. (2014) Isolation and characterization of polymorphic microsatellite loci from aerial yam (*Dioscorea bulbifera* L.). *Genetics and Molecular Research*, 13, 1514-1517.
